# Correlations Between Objective Behavioral Features Collected From Mobile and Wearable Devices and Depressive Mood Symptoms in Patients With Affective Disorders: Systematic Review

**DOI:** 10.2196/mhealth.9691

**Published:** 2018-08-13

**Authors:** Darius A Rohani, Maria Faurholt-Jepsen, Lars Vedel Kessing, Jakob E Bardram

**Affiliations:** ^1^ Embedded Systems Engineering Department of Applied Mathematics and Computer Science Technical University of Denmark Kongens Lyngby Denmark; ^2^ Copenhagen Center for Health Technology Technical University of Denmark Kongens Lyngby Denmark; ^3^ Copenhagen Affective Disorder Research Centre Psychiatric Centre Copenhagen Rigshospitalet Copenhagen Denmark; ^4^ Faculty of Health and Medical Sciences University of Copenhagen Copenhagen Denmark

**Keywords:** mood disorder, affective disorder, depression, depressive mood symptoms, bipolar disorder, objective features, correlation, behavior, sensor data, mobile phone, wearable devices, systematic review

## Abstract

**Background:**

Several studies have recently reported on the correlation between objective behavioral features collected via mobile and wearable devices and depressive mood symptoms in patients with affective disorders (unipolar and bipolar disorders). However, individual studies have reported on different and sometimes contradicting results, and no quantitative systematic review of the correlation between objective behavioral features and depressive mood symptoms has been published.

**Objective:**

The objectives of this systematic review were to (1) provide an overview of the correlations between objective behavioral features and depressive mood symptoms reported in the literature and (2) investigate the strength and statistical significance of these correlations across studies. The answers to these questions could potentially help identify which objective features have shown most promising results across studies.

**Methods:**

We conducted a systematic review of the scientific literature, reported according to the preferred reporting items for systematic reviews and meta-analyses guidelines. IEEE Xplore, ACM Digital Library, Web of Sciences, PsychINFO, PubMed, DBLP computer science bibliography, HTA, DARE, Scopus, and Science Direct were searched and supplemented by hand examination of reference lists. The search ended on April 27, 2017, and was limited to studies published between 2007 and 2017.

**Results:**

A total of 46 studies were eligible for the review. These studies identified and investigated 85 unique objective behavioral features, covering 17 various sensor data inputs. These features were divided into 7 categories. Several features were found to have statistically significant and consistent correlation directionality with mood assessment (eg, the amount of home stay, sleep duration, and vigorous activity), while others showed directionality discrepancies across the studies (eg, amount of text messages [short message service] sent, time spent between locations, and frequency of mobile phone screen activity).

**Conclusions:**

Several studies showed consistent and statistically significant correlations between objective behavioral features collected via mobile and wearable devices and depressive mood symptoms. Hence, continuous and everyday monitoring of behavioral aspects in affective disorders could be a promising supplementary objective measure for estimating depressive mood symptoms. However, the evidence is limited by methodological issues in individual studies and by a lack of standardization of (1) the collected objective features, (2) the mood assessment methodology, and (3) the statistical methods applied. Therefore, consistency in data collection and analysis in future studies is needed, making replication studies as well as meta-analyses possible.

## Introduction

Recently, there has been an increasing body of research investigating the use of mobile and wearable devices as a treatment intervention for depression [1]. Several mobile solutions have been proposed to utilize a self-monitoring and intervention-based treatment of depression [2-5]. One particular research approach adopted by many research groups has been to investigate how objectively measured behavioral features such as “location” and “social interaction” correlate with depression; using this approach, they have tried to differentiate euthymic and depressed states [6-11]. For example, using a mobile phone app passively recording information from sensors in the phone, Saeb et al [7] could show a statistically significant correlation between 6 different objective features, including mobile phone usage frequency and self-assessed mood using the Patient Health Questionnaire-9 (PHQ-9) scale [12] in nonclinical samples. Similarly, Faurholt-Jepsen et al [6] found 5 different objective features, including the number of outgoing short message service (SMS) text messages, which had a statistically significant positive correlation with depression severity as assessed using the Hamilton Depression Rating Scale (HDRS) in patients with bipolar disorder (BD).

The diagnostic process, as well as the process of symptom severity assessment in affective disorder, is based upon a combination of clinical evaluations and patient information, and there is a lack of objective markers of, for example, trait and state.

Digital behavioral markers have been defined as higher-level features reflecting behaviors, cognitions, and emotions, which are measured using low-level features and sensor data collected from digital technology, including mobile and wearable computing devices [13]. Many studies have found statistically significant correlations between objective behavioral features collected from mobile and wearable devices and mood symptoms in nonclinical samples of participants without psychiatric illnesses [14-17] as well as in clinical samples of patients diagnosed with psychiatric disorders [11,18-20].

The discovery of such significant correlations between objective features and depressive mood symptoms has raised great enthusiasm regarding using mobile and wearable devices in the treatment and monitoring of depression and other affective disorders. It has been argued that such an approach may provide an easy and objective way to monitor illness activity and could serve as a digital marker of mood symptoms in affective disorders [13,18]. Thus, if there is a well-established correlation between a specific digital marker—such as the number of steps taken and depressive mood symptoms—it would, in practice, be possible to develop an entirely automatic monitoring system. When, for example, the measured objective feature deviates from healthy behavior, an alarm or trigger could be raised in the clinic, which then could contact the patient [21].

However, when looking across individual studies, it is not easy to identify which objective features consistently correlate with depressive mood symptoms and in what way. Some studies have shown similar results, while others have shown contradicting results. For example, Beiwinkel et al [22] found a statistically significant negative correlation between the number of outgoing SMS text messages and the HDRS, whereas Faurholt-Jepsen et al [6] found a statistically significant positive correlation. Asselberg et al [15] found a negative correlation with mobile phone usage frequency and depressive symptoms, while Saeb et al [7] found the opposite.

No prior work has presented a comprehensive quantitative overview of objectively collected mobile features and how they relate to depressive mood symptoms. A more qualitative overview has recently been provided by Dogan et al [5], which highlights different mobile systems that have been developed to record subjective and objective features of individuals with affective disorders. They describe the findings of 29 different studies divided into different feature categories, such as physical activity, location, and phone usage, in a study-by-study evaluation.

Hence, a relevant question arises: to what degree studies show similar or different correlations between objective features and depressive mood symptoms, and how strong these correlations are? The purpose of this paper is to provide a systematic review of the available studies investigating the correlation between objectively collected features from mobile and wearable devices and depressive mood symptoms measured using various methods. Our systematic review aims to answer the following questions: (1) Which objective features have been collected? (2) What is the correlation between objective features and depressive mood symptoms? (3) Are the correlations similar across studies collecting the same features? Answering these questions could help us identify which objective features have shown most consistency across multiple studies and assist in designing future studies using technologies for objective assessment of depressive mood symptoms.

## Methods

### Systematic Review Process

We initiated the systematic review by following the PICO (Patient problem Intervention, Comparison, and Outcome) worksheet guidelines [23]. Then, we conducted and reported the systematic review according to the preferred reporting items for systematic reviews and meta-analyses statement [24].

### Inclusion and Exclusion Criteria

The following inclusion criteria were met with the included original papers: (1) The study involved any type of objectively measured features; (2) the data were collected via a mobile phone or other nonintrusive consumer-based mobile or wearable device; (3) participants were assessed on a mood scale, which included self-reported scales (eg, PHQ-9) or clinical diagnostic scales (eg, HDRS) used within psychiatry to quantify abnormal depressed mood either prior, during, or within the poststudy period; (4) comparisons of the objective features and the assessed depression scales between or within subjects were available or provided upon request from the respective corresponding author; (5) and as per the PICO Search Strategy, the following publication types were included: Meta-Analysis, Cohort study, Systematic Review, Case-Control Study, Randomized Controlled Trial, and Case series or report.

To ensure a broad inclusion of studies investigating the relationship between objective features and mood symptoms, the third statement was deliberately chosen to reflect a broad selection of clinical and nonclinical participants rated on different mood scales. This included both commonly used and clinically verified rating scales, such as the HDRS and PHQ-9, as well as nonstandard scales designed for a specific usage or technology, such as the 7-point (−3 to 3) scale used in the MONARCA (MONitoring, treAtment and pRediCtion of bipolAr Disorder Episodes) project [25,26].

We excluded original papers on the following premises: (1) nonquantitative studies or studies where only subjective features were collected; (2) if no English version of the paper was available; (3) studies that included participants with disorders other than mood disorders; (4) studies with nonhuman participants; (5) studies within social media since this topic has been thoroughly investigated elsewhere [27]; (6) studies with participants <18 years of age [28], to keep the focus on behavioral objective features collected on adults; (7) studies conducted before January 1, 2007; (8) studies that have not been published through peer review; and (9) the following publication types: trial protocols, in vitro or lab research, animal research, and editorials or letters or opinions.

### Search Strategy

The corresponding author (DAR) searched the following databases on November 25, 2016 to target both clinical and technical scientific literature: IEEE Xplore, ACM Digital Library, Web of Sciences, PsychINFO, PubMed, DBLP computer science bibliography, HTA, DARE, Scopus, and Science Direct. Systematic reviews and meta-analysis publications were included in the search for a subsequent cited reference search, which was conducted on April 27, 2017.

A broad database-specific search string was designed to target all studies that investigated mood disorders within a mobile setting. The specific search string for PubMed was as follows:

*(smartphone OR mobile OR wearable OR “smart phone” OR app OR apps) AND (depression OR bipolar OR unipolar OR “affective disorder” OR “mental health” OR “mood disorder”) AND (“2007/01/01”[Date–Publication]: “2017/01/01” [Date–Publication]) AND English[Language]*

The search strings for the other databases can be found in Multimedia Appendix 1.

The resulting publications were combined to one large spreadsheet, using an in-house Matlab script, with header information: database, title, author, publication year, publication type, and publisher.

### Study Selection

After removal of duplicates, studies were screened for eligibility in two phases. In phase 1, one author (DAR) excluded the studies based on the title. The title revealed several exclusion criteria, including different disorders (Alzheimer, schizophrenia, diabetes, chronic pain, autism, Parkinson, PTSD, or anorexia nervosa); nonhuman experiments; mobile phone addiction topics; focuses on diary methods, which only involve subjective data; use of internet-based interventions; and nonmedical-related topics such as bipolar electricity. In phase 2, one author (DAR) went through the abstract. If eligible, the full text was retrieved and reviewed. We excluded studies in which no objective features were collected, studies that only used self-assessment, and studies concerning emotion.

The resulting list, together with review papers from phase 1, were then used in a cited reference search by two authors (DAR, JEB) to produce the final list. The final list was critically investigated by all authors, which led to the exclusion of 16 papers due to outcome measures that did not represent mood assessment (eg, happiness scales [29-31], Quality of Life [32], or Satisfaction With Life Scale [33], as these do not reflect abnormal depressed mood) or wearables that were not consumer based (eg, a Holter monitor [34] or multisensory clothing [35-37]).

**Figure 1:**
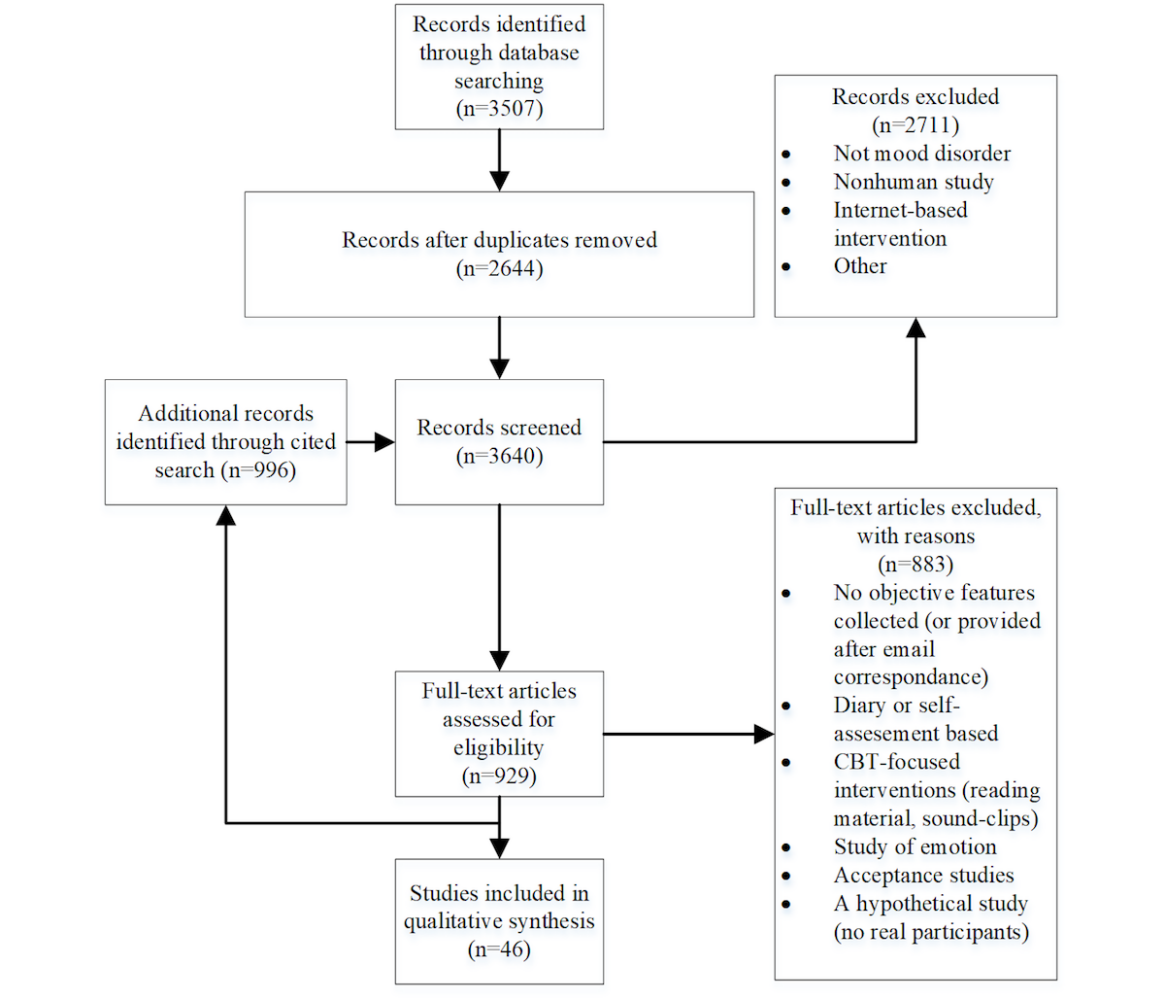
Flowchart illustrating the number of reviewed studies through the different phases. An exhaustive cited search was performed on the eligible studies, as represented by the “Additional records identified through cited search” box. CBT: cognitive behavioral therapy.

Several studies only reported correlation strengths or did not include correlation results between the objective features and the outcome assessment [9,14,31,38]. For these studies, we contacted the corresponding author via email and acquired the relevant data in all cases. The results of the study selection process are outlined in Figure 1.

### Data Extraction

Data were extracted from the final list by one author (DAR) in a predetermined format validated by a second author (JEB). The data were extracted into 2 separate tables; one for nonclinical samples of participants without psychiatric illnesses (Table 1) and one for clinical samples of patients diagnosed with Unipolar Disorder (UD) or BD (Table 2). The division into 2 tables was reviewed by all authors. Both tables listed the following data for each study; first author, year of publication, the specification of the mobile device, number of participants, participant age, days of the study, and the outcome depression scale. Table 2 included a diagnosis column. The supplementary material contains expanded versions of Tables 1 and 2 (found in Multimedia Appendices 2 and 4 respectively), which also include information about the method of recruitment and the method of assessing the relation between objective features and mood symptoms (eg, Pearson correlation, two-sample *t* test). The tables in Multimedia Appendices 3 and 5 provide a detailed overview of the different features for each study, classified into a feature category, the sensor used, a small description, and the results with respect to the mood assessment.

### Data Analysis

We were interested in investigating the correlation between behavioral objective features and depressive mood symptoms across all the included individual studies. To do this, we first identified all types of objective features, which have been applied in the eligible studies. The features were presented in a nomenclature list to create a standardized definition across all studies. Second, we investigated the strength of the correlation between objective features and depressive mood symptoms (ie, the *correlation coefficient*) across the included studies.

**Figure 2:**
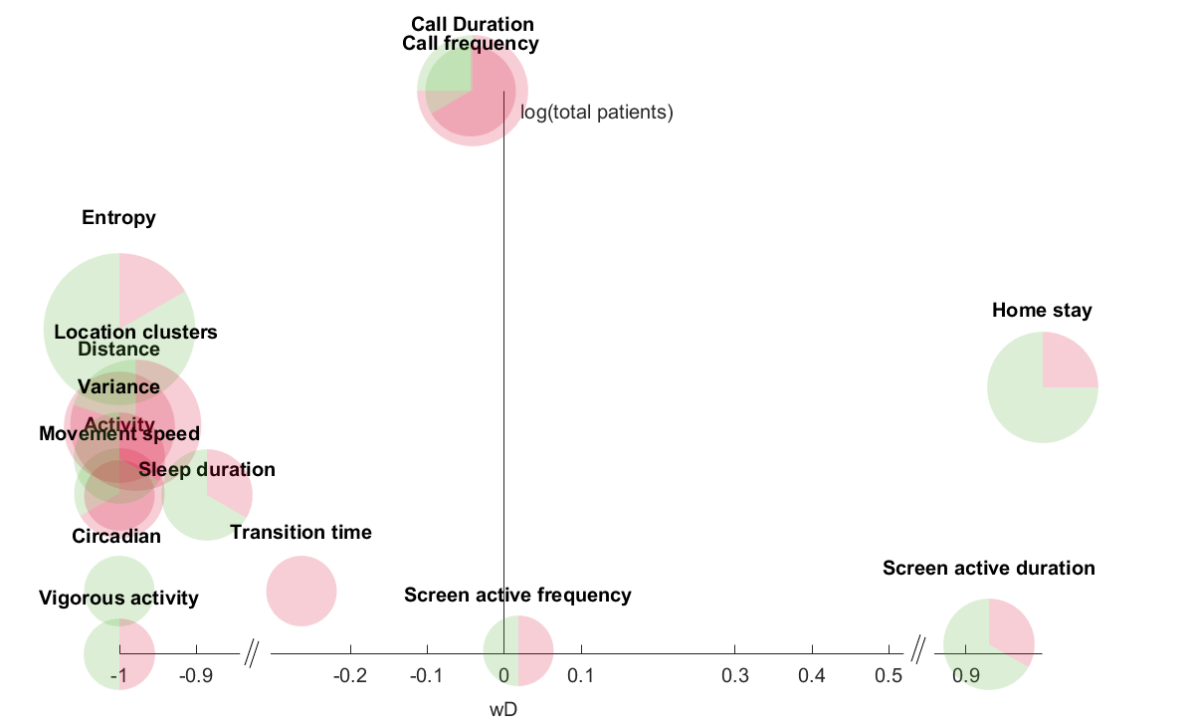
Features collected from at least two studies using nonclinical samples of participants. The x-axis (*wD*; weighted directionality) represents a weighted directionality of the correlation between the feature and mood symptoms. Positive values represent a larger depressive score and vice versa. The y-axis represents the logarithm of the total number of participants across all studies for this feature. The size of each pie chart represents the number of studies that recorded the feature, while the green, red, and gray areas represent statistically significant, statistically nonsignificant correlations, and missing statistical significance, respectively.

**Figure 3:**
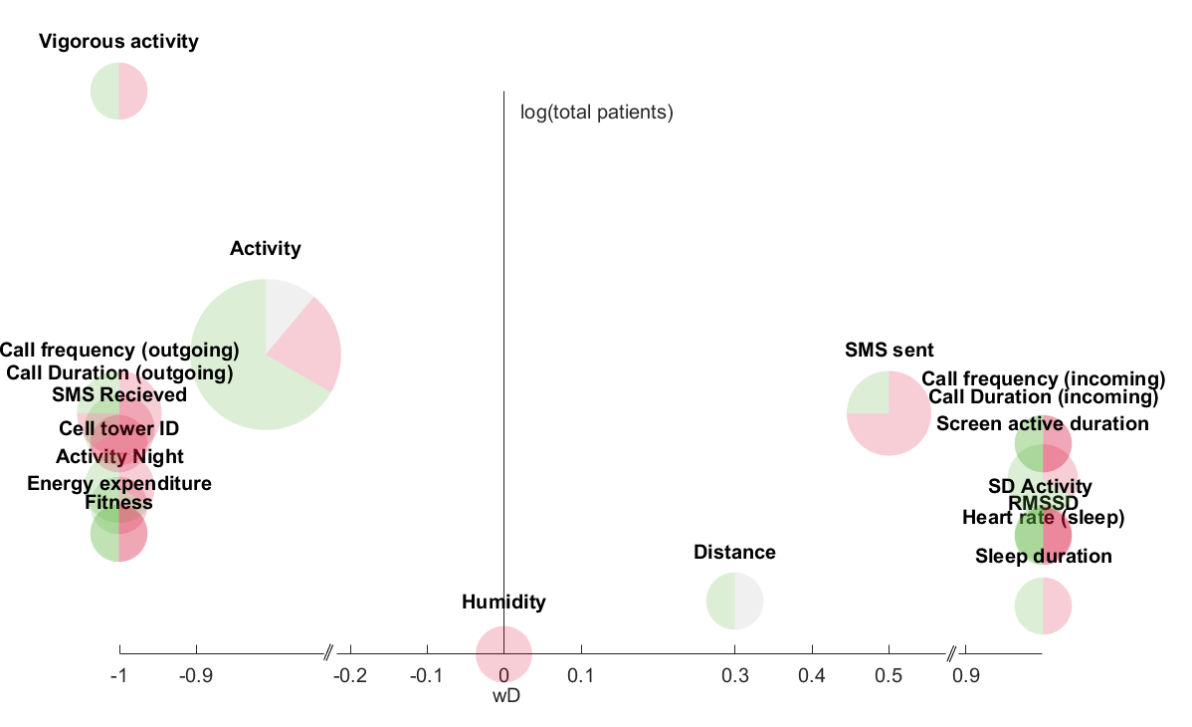
Features collected from at least two studies using nonclinical samples of participants. The x-axis (*wD*; weighted directionality) represents a weighted directionality of the correlation between the feature and mood symptoms. Positive values represent a larger depressive score and vice versa. The y-axis represents the logarithm of the total number of participants across all studies for this feature. The size of each pie chart represents the number of studies that recorded the feature, while the green, red, and gray areas represent statistically significant, statistically nonsignificant correlations, and missing statistical significance, respectively.

The investigation was performed by combining the directionality of the correlation values for identical objective features, weighted by the respective sample size and visualized as the x-axis and total sample size (log-transformed) on the y-axis. This was done in two separate graphs: one presenting nonclinical samples of participants (Figure 2 presents data from Table 1) and the other presenting clinical samples of patients diagnosed with either UD or BD (Figure 3 presents data from Table 2; Multimedia Appendix 6 shows patients with BD only). The two groups would most likely display different behaviors, and the separation was done on this premise. However, a combined result is displayed in Multimedia Appendix 7 for the convenience of the reader.

A positive directionality indicates that a larger quantity of the respective feature tends to give a higher depression score (eg, lower mood score, indicating a positive correlation with the depression score), while a negative directionality indicates that a larger quantity of the feature value tends to give a lower depression score (eg, a larger mood score, indicating a negative correlation with the depression score). All correlation values with outcome measures that represented larger values with better mood outcomes were multiplied by −1 to achieve the same weighted correlation directionality across studies.

**Table 1:** Summary of the included studies with nonclinical samples of participants.

Reference	Technology used	Participants (N=1189), n		Participant age (years), mean (SD)	Study duration (days)	Mood scale
		Male	Female			
Asselbergs et al, 2016 [15]	Android; Funf	5	22	21.1 (2.2)	36	10p mood
Baras et al, 2016 [40]	Android; EmotionStore	9	1	N/A^a^	14	BRUMS^b^
Becker et al, 2016 [41]	Android; Funf	5	22	N/A	42	Mood
Ben-Zeev et al, 2015 [42]	Android	37	10	22.5	70	PHQ-9^c^
Berke et al, 2011 [43]	Multisensor (waist)	4	4	85.3 (4.1)	10	CES-D^d^
Canzian and Musolesi, 2015 [9]	Android; MoodTraces	15	13	31	71	PHQ-8^e^
Cho et al, 2016 [44]	Phone records	234	298	57	N/A	BDI-21^f^
Chow et al, 2017 [45]	Android	35	37	19.8 (2.4)	17	DASS-21^g^
DeMasi et al, 2016 [46]	Android	17	27	N/A	56	BDI-21
Edwards and Loprinzi, 2016 [47]	Digi-Walker Pedometer	16	23	21.82	7	PHQ-9
Farhan et al, 2016 [17]	Android or iOS; LifeRhythm	21	58	18-25^h^	N/A	PHQ-9
Mark et al, 2016 [48]	Fitbit flex	20	20	N/A	12	Affect balance
Matic et al, 2011 [16]	Windows M. 6.5; MyExperience	6	3	28.4 (2.8)	7	rPOMS^i^
Mehrotra et al, 2016 [49]	Android	25^j^	N/A	N/A	30	PHQ-8
Mestry et al, 2015 [14]	Android	1	1	22	34	DASS21
Pillai et al, 2014 [50]	Actigraph	10	29	19.55 (3.2)	7	BDI-21
Saeb et al, 2015 [7]	Android; Purple robot	8	20	28.9 (10.1)	14	PHQ-9
Saeb et al, 2016 [39]	Android; Studentlife	38	10	N/A	70	PHQ-9
Wang et al, 2014 [51]	Android; Studentlife	38	10	N/A	70	PHQ-9
Wang et al, 2015 [52]	Android; Studentlife	37^j^	N/A	N/A	70	PHQ-9

^a^N/A: not applicable.

^b^Depression subscale of Brunel Mood Scale.

^c^PHQ-9: Patient Health Questionnaire-9

^d^CES-D: The Center for Epidemiological Studies Depression Scale.

^e^PHQ-8: Patient Health Questionnaire-8

^f^BDI-21: Becks depression inventory.

^g^DASS-21: Depression Anxiety Stress Scales.

^h^Study reported participant age as a range, rather than mean.

^i^rPOMS: reduced Profile of Mood States.

^j^Total number of participants; number of male and female participants not specified.

**Table 2:** Summary of the included studies with clinical samples of participants diagnosed with unipolar (UD) or bipolar (BD) disorder.

Reference	Technology used	Participants (N=3094), n		Clinical diagnosis	Participant age (years), mean (SD)	Study duration (days)	Mood scale
		Male	Female				
Abdullah et al, 2016 [53]	Android; MoodRhythm	2	5	BD	25-64^a^	28	SRM II-5^b^
Alvarez-Lozano et al, 2014 [11]	Android; Monarca	18^c^	N/A^d^	BD	N/A	150	7p mood
Beiwinkel et al, 2016 [22]	Android; SIMBA	8	5	BD	47.2 (3.8)	365	HDRS^e^
Berle et al, 2010 [54]	Actigraph	10	13	UD	42.8 (11)	14	Group difference
Dickerson et al, 2011 [55]	iOS; Empath	0	1	UD	83	14	10p mood
Doryab et al, 2016 [18]	Android	3	3	UD	>18^f^	20	CES-D^g^
Faurholt-Jepsen et al, 2012 [56]	Actiheart	8	12	UD	45.2 (12)	3	Group difference
Faurholt-Jepsen et al, 2015 [57]	Actiheart	7	11	UD	45.6 (11.1)	3	HDRS-17
Faurholt-Jepsen et al, 2016 [58]	Android; Monarca	9	19	BD	30.3 (9.3)	84	HDRS-17
Faurholt-Jepsen et al 2014 [10]	Android; Monarca	5	12	BD	33.4 (9.5)	90	HDRS-17
Faurholt-Jepsen et al, 2015 [26]	Android; Monarca	20	41	BD	29.3 (8.4)	182	HDRS-17
Faurholt-Jepsen et al, 2016 [6]	Android; Monarca	11	18	BD	30.2 (8.8)	84	HDRS-17
Gershon et al, 2016 [59]	Actigraph	14	23	BD	34.4 (10.4)	46	Group difference
Gonzales et al, 2014 [60]	Actigraph	15	27	BD	41.0 (11.2)	7	IDS-C-30^h^
Grünerbl; 2015 [61]	Android	2	8	BD	33-48	84	7p mood
Guidi et al, 2015 [20]	Android	0	1	BD	36	98	mood state
Hauge et al, 2011 [62]	Actigraph	14	11	UD	42.9 (10.7)	14	Group difference
Krane-Gartiser et al, 2014 [63]	Actigraph	5	7	BD	39.9 (15.6)	1	Group difference
Loprinzi and Mahoney, 2014 [64]	Actigraph (hip)	1261	1313	UD	46.3	7	Group difference
Miwa et al, 2007 [65]	Armband; SenseWear Pro	5	0	UD	35.1	87	Group difference
Muaremi et al, 2014 [66]	Android	6^c^	N/A	BD	18-65	76	7p mood
O’Brien et al, 2016 [8]	Actigraph	16	43	UD	74 (6)	7	MADRS^i^
Osmani et al, 2013 [19]	Android	0	5	BD	N/A	90	−3:3 mood^j^
Palmius et al, 2016 [67]	Android; AMoSS	9	27	BD	44 (14)	60	QIDS-SR16^k^
St-Amand et al, 2013 [68]	Actigraph	7	7	BD	44.6 (11)	14	Group difference
Todder et al, 2009 [69]	Actigraph	14	13	UD	49 (13)	7	Group difference

^a^Study reported participant age as a range, rather than mean.

^b^SRM II-5: Social Rhythm Metric II-5.

^c^Total number of participants; number of male and female participants not specified.

^d^N/A: not applicable.

^e^HDRS: Hamilton Depression Rating Scale.

^f^All participants in study above 18 years of age.

^g^CES-D: The Center for Epidemiological Studies Depression Scale.

^h^IDS-C-30: Inventory for Depressive Symptomatology, Clinical-rated.

^i^MADRS: Montgomery-Åsberg Depression Rating Scale.

^j^−3:3 mood: 7-point mood scale ranging from −3 to 3.

^k^QIDS-SR16: Quick Inventory of Depressive Symptomatology-Self Reported.

A meta-analysis of the specific correlation values was not considered for this systematic review. The heterogeneity across the studies was too substantial to perform any valid meta-analysis of correlations.

Not only were different analytical methods applied (eg, some using within-subject correlation others between subjects, some using day-averaged others week-averaged data) but also different apparatus and mood assessments were used. However, there is a clear correlation directionality invariance shown by studies comparing different analysis methods [6,22] and studies replicating same analysis methods on different datasets [7,39], which puts forth the argument that the directionality is a stable metric. Regarding the specific correlation values, we still encourage the reader to look at the results across studies using Multimedia Appendices 2,3,4, and 5 as a reference.

## Results

Of 3507 potentially eligible studies, 46 met the criteria of the review. A flowchart of the screening process is shown in Figure 1. Characteristics of the included studies are summarized in Tables 1 and 2.

Table 1 lists studies including nonclinical samples of participants (n=20), and Table 2 lists studies including clinical samples of patients diagnosed with either UD or BD (n=26). A more detailed overview of the included studies is listed in Multimedia Appendices 2,3,4, and 5.

We identified 7 overall behavioral feature categories, which we denoted as “Feature Categories.” These categories used 17 unique data inputs to analyze 85 different objective features. The same features were used across studies, yielding 176 investigated features, with information about directionality with respect to the mood score on 155/176 (88%) of the cases. The other cases (n=21) report on accuracy and weightings by combining objective features into single evaluations, which was mostly observed in research papers with classification models [53,61,66,67].

The 7 feature categories are defined and described in Table 3. An overview of the studies that contributed to each of the categories is provided in Multimedia Appendix 8. The supplementary files also include a graph illustration of the data inputs and how they contributed to the different category (Multimedia Appendix 9).

An in-depth analysis of each feature occurring in more than 2 studies is shown in Figures 2 and 3 for nonclinical and clinical samples of participants, respectively. Figures 2 and 3 were constructed as follows.

The x-axis is a weighted directionality of the correlation between the feature and mood symptoms. Positive values represent a larger depressive score and vice versa.(*wD*) is defined as:

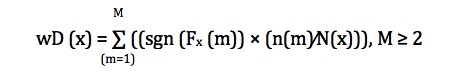



F_x_ is the correlation value of a unique feature such as *SMS text message sent*. M is the total number of F_x_ across all studies where N is the combined total number of participants. *“sgn”* denotes the sign operation which is −1 for values below zero, and 1 for values above zero. As an example, when considering the correlation between *screen active frequency* and mood symptoms, according to the table in Multimedia Appendix 3, this is analyzed in 2 studies; one study with N=28 shows a positive correlation, whereas one study with N=27 shows a negative correlation. This yields a *wD* as follows:

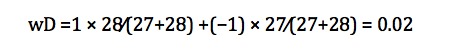



A *wD* value of 1 would indicate that all studies have a positive correlation between the measured feature and the mood assessment. This means that consistency across studies would place the feature on either +1 (consistent positive) or −1 (consistent negative) on the x-axis.

The y-axis is log-transformed values, to accommodate the large diversity, of the total number of participants on which the feature is measured. Nonclinical samples of participant studies measuring *call frequency* (n=370) had the highest average study participants, while clinical samples of participants measuring *humidity* (n=6) had the lowest.

The size of the feature pie chart represents *M*, which is the total number of studies of that particular feature. Similarly, the pie charts are divided into statistically significant (green), statistically nonsignificant (or lack of reporting; red) correlations, and missing information on statistical significance (gray).

In total, Figures 2 and 3 provide an overview of the correlation between statistically significant features and depressive mood symptoms. Each feature is followed by the result reported in the figure, which is the *wD* value, the number of studies that included the feature (n), the percentage of statistically significant cases (s), and the mean (SD) of the participants included in the “n” studies.

For nonclinical samples of participants (Figure 2), we observed the following:

Most studies, excluding *call duration* (*wD*=−0.04, n=4, s=25%, mean 278.50 [SD 293.32]), *call frequency* (*wD*=−0.04, n=3, s=33.33%, mean 370.67 [SD 279.44]), *screen active frequency* (*wD*=0.02, n=2, s=50%, mean 27.5 [SD 0.71]), and *transition time* (*wD*=−0.26, n=2, s=0%, mean 38.00 [SD 14.14]), agree on the correlation direction because most features are either at −1 or +1. *Home stay* (*wD*=1, n=4, s=75.00%, mean 56.75 [SD 23.32]), *circadian rhythm* (*wD*=−1, n=2, s=100%, mean 38.00 [SD 14.14]), and *entropy* (*wD*=−1, n=6, s=83.33%, mean 51.67 [SD 22.98]) have the largest number of statistically significant studies, whereas *distance* (*wD*=−1, n=4, s=0%, mean 45.75 [SD 24.10]), *movement speed* (*wD*=−1, n=2, s=0%, mean 63.50 [SD 21.92]), and *transition time* have no statistically significant studies.

**Table 3:** An overview of the included features together with the data input name separated into 7 distinct categories.

Feature Category	Feature
Social (n=38), with statistically significant correlation reported for 26% (10/38) results and statistical evaluation missing for 16% (6/38) results. Features describing social behavior, including activity related to phone calls, texting, social network size, and other people in the user's context.	Call duration (incoming or outgoing)-Call log Call frequency (incoming or outgoing)-Call log Calls missed-Call log Maximum call duration-Call log Number of conversations-Call log SMS^a^ text messages received (characters)-SMS text message log Characters in SMS text message (sent or received)-SMS text message log SMS text message (sent or received)-SMS text message log Speak duration-Call log Devices seen-Bluetooth
Physical activity (n=48), with statistically significant correlation reported for 46% (22/48) results and statistical evaluation missing for 6% (3/48) results. Features describing physical activity, including movement and step count.	Activity (afternoon, day, evening, morning, night)-Accelerometer Autocorrelation-Accelerometer Vigorous activity-Accelerometer Distance-Accelerometer, GPS^b^ Energy expenditure-Multiple sensors Fourier analysis-Accelerometer Inactivity duration-Accelerometer Jerk-Accelerometer Movement duration-GPS Movement speed-Accelerometer, GPS Movement speed variance-GPS RMSSD-Accelerometer Sample Entropy-Accelerometer SD of stillness-Accelerometer Steps-Accelerometer, Pedometer
Location (n=38), with statistically significant correlation reported for 50% (19/38) results and statistical evaluation missing for 8% (3/38) results. Features describing mobility, including GPS tracking, clustering of location (eg, home stay), and transition time.	Cell tower ID-GSM^c^ Home stay-GPS Location clusters-GPS Break duration-FM radio signal Circadian rhythm-GPS Entropy-GPS Home to location cluster-GPS Maximum distance between clusters-GPS Raw entropy-GPS Routine index-GPS Transition time-GPS Location variance-GPS Coverage area-GPS
Device (n=24), with statistically significant correlation reported for 54% (13/24) results and statistical evaluation missing for 0% (0/24) results. Features describing device (mobile phone or wearable) usage, including app usage, lock or unlock events, and classification of app usage.	Communication or social usage-App Duration-App Browser usage-App Images taken-Camera Number of running apps-App Response time-Notification Screen active duration or frequency-Screen Screen clicks-Screen Time from arrival till seen-Notification Time from seen till acted-Notification Data transmitted-Wi-Fi
Subject (n=24), with statistically significant correlation reported for 50% (12/24) results and statistical evaluation missing for 21% (5/24) results. Features capturing the subject's physical state, including sleep and voice.	Deep sleep or total sleep-Accelerometer Deviation of F0-Microphone Envelope-Microphone Fitness-ECG^d^ Fundamental frequency-Microphone Harmonics-to-noise ratio-Microphone Pauses in recording-Microphone Short turns during conversation-Microphone Sleep (duration, efficiency, onset latency)-Accelerometer SD pitch frequency-Microphone Laying down-Camera SD sleep-Accelerometer
Environment (n=2), with statistically significant correlation reported for 0% (0/2) results and statistical evaluation missing for 0% (0/2) results. Features collected from the physical surroundings of the user.	Intensity level-Light sensor Humidity-Internet
Bio (n=2), with statistically significant correlation reported for 50% (1/2) results and statistical evaluation missing for 0% (0/2) results. Biometric features related to the subjects body.	Heart rate (sleep, day)-LED^e^ light sensor, ECG Skin conductance-EDA^f^

^a^SMS: short message service.

^b^GPS: global positioning system.

^c^GSM: Global System for Mobile communication.

^d^ECG: electrocardiography.

^e^LED: light-emitting diode.

^f^EDA: electrodermal activity.

Similarly, for the clinical sample of patients (Figure 3), we observed the following:

Most studies, excluding *distance* (*wD*=0.30, n=2, s=0%, mean 10.00 [SD 4.24]), *humidity* (*wD*=0, n=2, s=0%, mean 6.00 [SD 0.00]), *SMS text message sent* (*wD*=−0.50, n=4, s=25%, mean 30.00 [SD 21.76]), and *activity* (*wD*=−0.81, n=9, s=66.67%, m 23.33 [SD 16.10]), agree on the correlation direction because they are at either −1 or +1 on the *wD* axis. *Cell tower ID* (*wD*=−1, n=3, s=66.67%, mean 19.67 [SD 8.33]), *screen active duration* (*wD*=1, n=3, s=66.67%, mean 21.33 [SD 6.66]), and *activity* have the largest statistically significant percentage, whereas *distance*, *SMS text message received* (*wD*=−1, n=2, s=0%, mean 45.00 [SD 22.63]), and *humidity* have the lowest.

Several objective features were only included in a single study. Therefore, their relationship to a depressive mood scale cannot be compared across studies as done in Figures 2 and 3. Some of these features are quite creative and worth mentioning. The most promising results for the nonclinical samples include the time spent in break rooms (ρ=−0.21, nonsignificant) [16], and less SD of stillness amount, which can be interpreted as a more uniform activity pattern (beta=−3.3, *P*<.001) [46]. For the clinical samples, it includes the increased amount of time with no sound detection (*speech pauses*; beta=0.34, *P*=.004) [55], increased number of *calls missed* (beta=0.05, *P*=.006) [6], and fewer incidences of quick or sudden movements (*jerk*; *t*=4.06, *P*<.001).

### Data and Methods Reporting

In the 46 eligible studies, 19 different mood assessment methods were used. The most common assessment method was the PHQ (n=9), whereas assessment methods like the Montgomery-Åsberg Depression Rating Scale and the Brunel Mood Scale [40], which are patient-reported outcome measures, were only used in a single study.

Seven different technologies were used for collecting the objective features. The most frequent one was mobile phone (n=30); mostly Android phones were used (n=27), with iOS (n=2) and Windows (n=1) phones also being used. Wearable devices were reported to be located on various areas on the body, including the upper arm [65], wrist (nondominant hand [69], right hand [62]), waist [43], hip [64], and chest [57].

When analyzing the relation between the objective features and depressive mood assessments, we identified several regression-, machine learning–, correlation-, and group-difference methods. In total, across the 46 eligible studies, 12 different methods were used, with Pearson correlation being the most used (n=17).

Details on the analysis method were, in general, not well documented. This especially applied to studies where correlation analysis was secondary to the main hypothesis [70,71]. Important details that were mentioned in few of the studies included possible confounding variables such as age, sex, and body mass index (BMI) [18,53]; data sampling methods such as global positioning system (GPS) polling strategies [30]; the window-length in days or averaging methods of objective data that were correlated with the outcome [7,51]; and within-subject or between-subject analysis [22,42]. Full transparency, by providing the data, was seen in only 2 of the studies [15,51], with 5 studies using existing public data [39,64,70-72].

## Discussion

### Principal Findings

In this paper, we present the results of the first systematic review on the correlation between objective behavioral features collected via mobile and wearable devices and the assessment of depressive mood symptoms as measured by different rating scales and questionnaires. This was possible due to the increased research on mobile and wearable computing devices in the context of mental health [4,73,74], yielding 46 included studies in this review. We found that 57% (26/46) studies (a small majority) were performed on clinical samples of participants. However, when analyzing the number of participants included in these studies, they constituted a majority (3094/4283, 72%). We separated these two groups since nonclinical samples of participants are, by definition, healthy and, most likely, will display different behaviors than clinical samples of patients diagnosed with UD or BD.

We want to emphasize, for the subsequent discussions, that correlation assessments do not imply causality, but rather simple associations. The correlation between two measures could also be mediated through one or several covariates, which were not explored in any of the included studies [75]. For instance, Disabato et al were able to validate a correlation by including a statistical mediation model [76]. They concluded that the presence of positive life events mediated the correlation between gratitude and depression. A simple correlation assessment also does not provide knowledge on the clinical utility of these data in the classification of affective episodes in UD or BD because sensitivity, specificity, positive predictive values, and negative predictive values were not investigated in most studies. However, discovering and understanding the relationships between objective features and their relation to mood symptoms may be relevant in a clinical setting because it may provide an easy and objective way to monitor illness activity outside the clinical settings and could serve as a digital marker for mood symptoms [18].

### Feature Categories

The *social* category had the lowest percentage of statistically significant correlations, by vote counting, across studies (10/38, 26%). *Social* included features such as *call duration* and *number of conversations*, which can be accessed on Android phones, contrary to iPhones [77]. We did not find any research article that explains how social patterns change with depression, but the review article by Baker et al [27] on online social networks suggests a complex relation involving factors that mediate or moderate the correlation and increase the variability in the findings. Furthermore, Cho et al [44] found a direct opposite correlation between genders (male negative, female positive) in the *call duration* and *call frequency* features. This suggests that social-based features should be treated as a highly personalized feature that should be assessed in a within-subject analysis.

The feature category with the highest percentage of statistically significant correlation features across studies was *device* (13/24, 54%). As an example, using data provided by the corresponding author [14], we observed statistically significant results in *communication app usage* (*r*=−0.33, *P*=.007) calculated using a within-subjects analysis of covariance. The low variability with device-based features could indicate that there is a general tendency for participants to use their phones more, but at the same time, withdraw from the social context by lowering the *communication app usage*.

The feature category *subject* is similar to *Device*, investigated less but with a high percentage of statistically significant correlations across studies (12/24, 50%). This includes features within sleep and voice. In particular, *sleep duration* was the most investigated feature (n=6), with statistically significant correlations in 4 studies Furthermore, *subject* was one of the less included categories, which could be due to the second-level processing required to achieve features of voice [66] or sleep durations through multiple sensors [51].

### Objective Features

#### Nonclinical Samples of Participants

As seen in Figure 2, we found two features that have a strong positive (ie, close to 1 *wD*) correlation with depression: *home stay* and *screen active duration*; both of these showed a large proportion of statistically significant correlations across studies. Moreover, all 4 studies with a positive correlation between *home stay* and depression level also had a large average participant number. Individual studies have shown that the degree to which a person stays at home is associated with depression [45], and it is a general hypothesis that this relation is positive. We were able to verify this hypothesis by combining the results across the included studies in this review.

On the other hand, no prior hypothesis has been formulated regarding the relationship between general phone usage and depressive mood symptoms. However, studies have shown a statistically significant positive correlation between depressive symptoms and the feature *screen active duration* [78]. Similarly, subjective-based mobile phone use has been studied in relation to depression, where Thomée et al found that high mobile phone use was associated with symptoms of depression [79]. These findings were replicated in this review, with only a single statistically nonsignificant contradictive result from a two-sample study by Mestry et al [14] (*r*=−0.03, *P*=.79).

On the left side in Figure 2, we see several features that have a strong negative correlation to depression, including *location clusters*, *entropy*, and *sleep duration*. A majority of these features indicates that enhanced physical activity and more movement outside of the house are observed when participants score lower on the depression scale. This is consistent with the Actigraph systematic review papers by Scott et al [80], who revealed a consensus of lower mean activity levels associated with bipolar depression, and Burton et al [81], who revealed a pattern of lower daytime activity but higher nighttime activity in depression.

*Entropy* is the most prominent feature in the figure with many studies (n=6), all yielding a negative correlation and a high statistically significant proportion. The only case of nonsignificance was reported by Saeb et al [7] (*r*=−0.42, *P*=.082), who, however, did show a high negative correlation. *Entropy* is a measure that captures the distribution of time spent at the different location clusters registered. Thus, a high *entropy* would indicate that the participant spends time more uniformly across different location clusters. Because all studies consistently showed a negative correlation, this implies that a higher *entropy* correlates with a better mood. If a participant stays home for a longer time than usual, the *entropy* will drop. Hence, there is a dependency between *entropy* and *home stay*, which is also evident in the figure where they are almost mirrored, both with a large proportion of statistically significant findings. Both features can be collected via the location Application Programming Interface, which uses the GPS sensor typically embedded in all mobile phones or wearables.

Features with less consistent findings across studies regarding positive or negative correlations are located closer to 0 *wD*; these include features such as *screen active frequency*, *call duration*, *call frequency*, and *transition time*. At first look, it seems that these features are not related to mood symptoms and, hence, exhibit random correlation values. However, another explanation could be gender or cultural differences. In a cross-cultural study with people from Switzerland and Turkey, Hernández et al [29] found different correlation directions between the two groups in *screen active frequency* and *number of running apps*. Furthermore, several device-based features such as *browser app usage* and *reading app usage* have different correlation directions between genders (male positive, female negative) [33], and the two social features *call duration* and *call frequency* also exhibit different correlation directions between genders (male negative, female positive) [44].

*Transition time* has been currently only investigated by the research group of Saeb et al [7,39], who conducted a study to replicate previous findings of the same features. The first study showed a positive correlation (*r*=0.21, *P*=.40), while a second study showed negative correlation (*r*=−0.32, nonsignificant). The feature then yields a low negative *wD* due to the latter including more participants and placed more centrally due to the contradictive results.

#### Clinical Samples of Patients Diagnosed With Unipolar or Bipolar Disorder

The feature *screen active duration* is similar to the nonclinical samples, with a high proportion of statistically significant studies and a consensus on positive correlation among the studies. Note, however, that this feature was the only one within the *Device* category that was investigated for both nonclinical and clinical samples of participants.

The features of *sleep duration* and *distance* have switched to a positive *wD* in Figure 3 compared with Figure 2. Only 2 studies have investigated *distance* for clinical samples of patients. Beiwinkel et al [22] reported a negative correlation in a between-subject analysis, but the within-subject analysis that we reported had almost zero correlation (*r*=0.03, *P*=.66). In contrast, Abdullah et al [53] showed a negative correlation direction by the negative weighting coefficient (w=−1.56 × 10^−2^) using the Support Vector Machine analysis. However, with a small number of total participants, only 2 studies, both nonsignificant, *distance* was found to be weakly represented in the literature. *Sleep duration*, on the other hand, had statistically significant findings in both groups. This feature is a good example of the reasoning in analyzing depressed symptoms in clinical samples separately from the nonclinical sample. In clinical samples of nonseasonal depression, patients often suffer from abnormal sleep patterns with problems falling asleep, interrupted sleep, and early morning waking, while such a sleep pattern not is seen among healthy subjects.

Social-based features were more extensively investigated with clinical samples of patients. The two features *incoming call duration* and *incoming call frequency* reveal a strong tendency that participants tended to receive more calls and talk longer during these calls when depressed. On the other hand, the features *outgoing call duration* and *outgoing call frequency* tend to suggest that patients make more and longer calls when they are less depressed. This difference between incoming and outgoing calls highlights that these features should be kept separate, and it raises concerns with some of the results on *Call duration* with nonclinical samples of participants as in a study by Wang et al [51], who measured *call duration* and *frequency* across incoming and outgoing calls.

The feature *SMS text messages sent* was found to have a lower *wD*, showing inconsistencies across the 4 studies. We did not find any results in the literature that could explain the lower *wD* on *SMS text messages sent*, although the use of internet- and app-based chat and video communication platforms has been increasing, while SMS text message communication has fallen drastically. In Denmark, there has been a drop of 19.6% in SMS text messages sent from 2015 to 2016 [82]. This suggests that SMS text message logging should be used with caution and should be extended to include other relevant messaging technologies.

The clinical sample consisted of both unipolar and bipolar patients. Optimally we would have liked to analyze data separately for these two patient groups due to findings that show psychomotor activity and sleep discrepancies between unipolar and bipolar depression [80]. However, the focus here is on the level of depressive mood symptoms as a function of objective features, where bipolar and unipolar patients show same directionality compared with healthy controls [56]. Nevertheless, we repeated the analysis of correlation directionalities, including patients with BD only, and the results remained unchanged. See Multimedia Appendix 6.

### Limitations

#### Data Collection and Analysis Method

When combining the studies investigating objective features and their relation to mood symptoms, it became apparent that a meta-study on the exact correlation values would be misleading. The lack of detailed reporting on analysis methods was clearly demonstrated in a study by Beiwinkel et al [22], where a between-subject (cross-sectional analysis) relationship yielded a statistically nonsignificant (*P*=.82) regression coefficient of −0.04, while a within-subject (longitudinal analysis) relationship yielded a statistically significant (*P*=.03) regression coefficient of −0.11, on the feature of *cell tower ID.* Data aggregation length was also a concern because the duration of studies included in this review spans from 7 days [16,47] to 12 months [22]. Canzian & Musolesi [9] presented results on the correlation between PHQ-8 and different mobility features for 1 to 14 days of aggregation. The absolute correlation value increased from .152 (−.016 not absolute) to .432 on the feature *maximum distance*. The change was most likely due to a larger data pool, which lowered the variance toward “outlier” days or even noise in the data stream. It might also be related to the day of the week. For instance, Saeb et al [39] found variations in the objective feature *home stay* between work days and weekends. Furthermore, the lack of reporting confounding variables in the analysis was a concern. Faurholt-Jepsen et al [6] have demonstrated the effect of adding confounding variables to the analysis, where an unadjusted model without confounding variables on, for example, *screen active duration* (beta=194.8, *P*=.06) becomes statistically significant when controlling for age and sex (beta=209.6, *P*=.04).

To investigate depressive severity, many studies measured mood pre-, during, and poststudy, and there were correlation differences depending on when the mood assessment was done [51]. In a more detailed study [39], we saw a gradual lowering in correlation between various objective features and prestudy PHQ-9, which was not that surprising because the PHQ-9 questionnaire captures symptoms of the last 2 weeks and not future behavior. However, interestingly, the correlation was stable in the 8 weeks when the features were assessed using the poststudy questionnaire. Ben-Zeev et al [42] looked even closer on a day-based sample resolution. Here we see a directionality switch with some of the objective features. Sleep duration was modeled with a positive regression coefficient with pre-post change PHQ-9 (eg, higher sleep duration modeled a worse PHQ-9 change score) almost throughout the study period, but during the last quarter, it changed to a negative regression coefficient, which is consistent with the literature, as depicted in Figure 2 [48,51]. These findings highlight the importance of transparency regarding the analysis methods. The implications regarding the results presented in this systematic review are minimal because the induced correlation differences remain invariant to the correlation directionality, which is the focus here.

Limitations are also associated with the different technologies, including hardware and software, used to collect objective features from mobile phones and wearables. Studies have shown statistically significant differences that need to be accommodated within the study design [83]. For example, Farhan et al [17] developed a mobile phone-based sensing app with the PHQ-9 assessment on both iOS and Android. The study showed that the feature *movement duration* changed from a correlation of *r*=0.06 (*P*=.43) on Android to *r*=−0.13 (*P*=.07) on iOS. They argued that the difference was due to technical details regarding whether data was pooled or sent from the sensor. This example demonstrates a change in correlation directionality, which could have had an impact on our results if more studies were reporting on movement duration. Even though we reported the Android results, to be consistent with the other mobile phone-based studies, there was no impact on our results because the directionality of the remaining features was identical.

The result on weighted directionality in Figure 3 includes 9 studies that reported on group differences between clinical subjects and healthy controls. They compared the mean value of the feature between the two groups to understand what directionality the feature has concerning the disorder. If the study reported longer sleep duration in the clinical group than in healthy controls, it indicates that the directionality is positive. This could be problematic in nonlinear cases, such as the observation by McKercher et al [84]. They found that male participants with depression were taking 7500-9999 steps per day, contrary to healthy controls who were in the lower or upper levels of respectively <7500 or >9999 steps per day.

#### Mood Assessment

The included studies used different ways of measuring mood symptoms, which undoubtedly had an impact on the correlation value, while the directionality of the correlation stayed intact. Several studies have shown a high correlation between different mood assessment methods. Simple mood scales for self-assessment, such as a 7-point selection from −3 till 3, have shown statistically significant correlations with clinically validated rating scales such as the HDRS [26]. For example, there is a high correlation between the commonly used assessments methods of depression; PHQ-9, Becks depression inventory (BDI), and HDRS (lowest PHQ-9 vs HDRS: *r*=0.73; Table 3) [85]. The Center for Epidemiological Studies Depression Scale and BDI have also been shown to be highly correlated (*r*=0.84, *P*<.001) [86]. Patient-based outcome measures such as PHQ-9 and BDI have the benefit of being conducted outside the clinic, target very specific symptoms, exclude clinician bias, and facilitate the doctor-patient communication. However, they have some drawbacks such as a biased response depending on the recipient and a lack of meaningful interpretation of the changes to the outcome value [87].

As previously mentioned, we have chosen to include a broad definition of mood-based assessments in this review. However, a limitation is that some of them are questionable in the assessment of mood and depression. For instance, studies assessing “happiness,” “well-being,” and “quality of life” have been excluded in this review [29-33], even though it has been shown that happiness scores correlated moderately with depression, measured using BDI (*r*=−0.57, *P*<.001) [88].

The heterogeneity of the included studies also limits implementations in future studies. Faurholt-Jepsen et al [6] presented a new feature *calls missed*, which is statistically significantly correlated with HDRS-17 (beta=0.05, *P*=.006). The result was presented in 2016, but not replicated in any of the later studies, such as the comprehensive study on phone records with 532 subjects [44].

#### Absolute Valued Correlations

Several research groups chose to present their correlation results in absolute values [9,49,89]. This is a problem because the directionality of the correlation is lost, and the only information left is a measure of the strength of the relation. Canzian & Musolesi [9] clearly visualized this problem in several histogram plots representing each subject correlation values; these plots almost resemble a normal distribution around zero, but with a tail toward one of the directions. Raw correlation values were provided when requested from 1 of 3 studies [9]. The other 2 commented on their choice of reporting absolute values of the correlation:

We observed very different behaviors among users, having in some cases positive correlations, in others, negative ones and in others no correlation at all [89].

However, as Figures 2 and 3 show, there are several consistent correlations between features and mood assessments. Therefore, because this systematic review has revealed several features with common correlations across multiple studies, we hope to encourage future studies to present raw correlation values. This will make cross-study comparisons more valid. Further discussion on the use of absolute correlation values can be found elsewhere [90].

### Future Directions

The analysis provided in this paper has shown that it is time consuming and difficult to compare and analyze data across studies due to a high level of heterogeneity. To provide more systematic and automatic analyses, a significant degree of standardization is needed in three areas:

1. Standardized data collection and feature extraction. The way that physical activity, social activity, and mobility features based on accelerometer and GPS data are extracted should be standardized across studies. For example, the feature *location entropy* seems like a promising feature and could be collected and calculated consistently across studies.
2. Standardized mood assessment tools. The review revealed that a wide range of clinical (n=11) and nonclinical (n=9) mood rating scales were used. This makes it hard to compare correlations across studies when such different scales are used. We suggest that future studies include a clinician-based rating scale of severity of depression such as the HDRS as well as a self-reported questionnaire of depression such as the PHQ-9 or the BDI-21.
3. Standardized statistical correlation methodology. The reviewed papers applied more than 11 different methods for correlation values, with different time windows. We suggest that raw correlation values are presented in addition to associations adjusted for relevant demographic variables, including sex and age, and clinical variables, such as BMI.

We also invite future systematic reviews to focus on classification models. They include accuracy measures and weightings that assist in the understanding of the individual objective features to classify mood and can investigate nonlinear interactions between multiple features and mood scores. As an example, Muaremi et al [66] used microphone features to classify mood; they achieved an F1 accuracy of 82% and discovered *speaking time* as the best-performing feature. By expanding to include GPS and accelerometer-related features, Abdullah et al [53] achieved an F1 accuracy of 85.5%, with the GPS feature *distance* achieving largest weighting. A Naïve Bayes Classifier, to predict mood based on a combination of location features, achieved an accuracy of 81.7% [61].

In our search, we came across several studies with sensor systems that are not currently fully mobile. This includes electroencephalogram (EEG) systems [91-93]. For instance, Li et al [93] achieved a 99.1% accuracy discriminating depressed and nondepressed participants based on EEG. Other systems monitoring body temperature [94], saliva [95], autonomic nerve balance [96], and facial muscle activities [97] could also be relevant. However, because these sensor modalities are not mobile or wearable to any great extent, they were excluded. These sensor modalities could, however, potentially be included in a ubiquitous mobile system for mood disorders in the future.

### Conclusions

Mobile and wearable devices provide a unique platform for continuous collection of behavioral data from patients in real-time and within naturalistic settings. Many researchers have used this to investigate the relationship between behavior and mood disorder symptoms, as recorded by mobile or other wearable devices. In this systematic review, we identified a total of 46 eligible papers of such studies, of which 26 involved clinical samples.

We found 7 feature categories (Table 3) that were investigated across the studies. Subject-based and device interaction features represented the largest percentage of statistically significant relationships. In a detailed analysis of the 85 objective features that were identified, we were able to find strong consistencies between several behavioral features across the studies. For example, in the nonclinical sample, there was a consistent positive correlation between the features *home stay* and mobile phone *screen active duration* with mood symptoms (eg, more time at home and longer phone usage indicated a more depressed mood). Furthermore, several behavioral features had a coherent negative correlation with mood symptoms, including amount of *vigorous activity*, *location variance*, and *distance* moved. In the clinical samples, mobile phone *screen active duration* was replicated as a constant positive correlating feature together with *incoming call frequency* and *duration*. Similarly, a coherent negative correlation was found, including the amount of visible *GSM cell towers* (reflecting mobility), *SMS text messages received,* and *outgoing call frequency* and *duration*.

## Appendix

Multimedia Appendix 1 Search string used in all the database searches.

Multimedia Appendix 2 Extended information of the included study for the nonclinical samples.

Multimedia Appendix 3 The reported results between the measured objective feature and the outcome measure for the nonclinical sample.

Multimedia Appendix 4 Extended information of the included study for the clinical sample.

Multimedia Appendix 5 The reported results between the measured objective feature and the outcome measure for studies with a medical diagnose of either Unipolar Disorder (UD) or Bipolar Disorder (BD).

Multimedia Appendix 6 Features collected from at least two studies using clinical samples with Bipolar disorder only. The x-axis (wD) represents a weighted directionality of the correlation between the feature and mood symptoms. Positive values represent a larger depressive score and vice versa. The y-axis represents the logarithm of the total number of participants across all studies for this feature. The size of each pie chart represents the number of studies that recorded the feature, while the green, red, and grey areas represent statistically significant, statistically nonsignificant correlations, and missing statistical significance respectively.

Multimedia Appendix 7 Features collected from at least two studies using clinical and nonclinical samples of participants. The x-axis (wD) represents a weighted directionality of the correlation between the feature and mood symptoms. Positive values represent a larger depressive score and vice versa. The y-axis represents the logarithm of the total number of participants across all studies for this feature. The size of each pie chart represents the number of studies that recorded the feature, while the green, red, and grey areas represent statistically significant, statistically nonsignificant correlations, and missing statistical significance respectively.

Multimedia Appendix 8 Table of the 46 included studies showing the feature category that they report on.

Multimedia Appendix 9 Graphical linkage showing which sensor types are used within the seven feature categories.

## Abbreviations

BD: bipolar disorder
BDI: Becks depression inventory
BMI: body mass index
CBT: cognitive behavioral therapy
EEG: electroencephalogram
GPS: global positioning system
HDRS: Hamilton Depression Rating Scale
MONARCA: MONitoring, treAtment and pRediCtion of bipolAr Disorder Episodes
PHQ: Patient Health Questionnaire
PICO: Patient problem Intervention, Comparison, and Outcome
SMS: short message service
UD: unipolar disorder
