# Supplementary material for: Correlations Between Objective Behavioral Features Collected From Mobile and Wearable Devices and Depressive Mood Symptoms in Patients With Affective Disorders: Systematic Review
Source: JMIR Mhealth Uhealth. 2018 Aug 13;6(8):e165. doi: 10.2196/mhealth.9691 (PMC6111148; doi:10.2196/mhealth.9691)
Supplement: Multimedia Appendix 1 [file mhealth_v6i8e165_app1.pdf]

Search terms for:

PUBMED:

Search **(smartphone OR mobile OR wearable OR "smart phone" OR app OR apps) AND (depression OR bipolar OR unipolar OR "affective disorder" OR "mental health"[Text Word] OR "mood disorder”) AND ("2007/01/01"[Date - Publication] : "2017/01/01"[Date - Publication]) AND English[Language])**

ACM (ACM Guide to Computing Literature):

"query": { ("smartphone" OR "mobile" OR "wearable" OR "smart phone" OR "app" OR "apps") AND ("depression" OR "bipolar" OR "unipolar" OR "affective disorder" OR "mental health" OR "mood disorder") }

"filter": {"publicationYear":{ "gte":2006, "lte":2016 }},

{owners.owner=HOSTED}

Web of Science:

(TS=(smartphone OR wearable OR "smart phone") AND TS=(depression OR bipolar OR unipolar)) *AND* **LANGUAGE:** (English)
**Timespan:** 2007-2016.

Psychinfo:

Search for: Any Field: smartphone OR mobile OR wearable OR "smart phone" OR app OR apps AND Any Field: (depression OR bipolar OR unipolar OR "affective disorder" OR "mental health" OR "mood disorder") AND Year: 2007 TO 2017

Database(s): PsycINFO, PsycBOOKS, PsycCRITIQUES, PsycARTICLES, PsycEXTRA

DBLP Computer Science Bibliography:

(Since nothing means “*”, tab = OR, space = AND)

<query id="238196">smartphone*|mobile*|wearable*|phone* depression*|unipolar*|bipolar*|mood* </query>

SCOPUS (Elsevier)

( ( smartphone OR wearable OR "smart phone" OR app OR apps ) AND ( depression OR bipolar OR unipolar OR "affective disorder" OR "mental health" OR "mood disorder" ) ) AND PUBYEAR > 2006

Science Direct

Search results: 289 results found for pub-date > 2006 and (smartphone or mobile or wearable) AND (depression or unipolar or bipolar or "mood disorder") AND LIMIT-TO(cids,

"271802,271071,271442,271031,271069,271189,271821,271078,271035,271080,271077","Computers in Human Behavior,Neuroscience,Journal of Pharmaceutical and Biomedical Analys...,Behavioural Brain Research,Neuropharmacology,International Journal of Pharmaceutics,Social Science & Medicine,European Neuropsychopharmacology,Journal of Affective Disorders,Brain Research,European Journal of Pharmacology") AND LIMIT-TO(topics, "internet,social,patient,health,mental health,depression,bdnf,hplc,poster session,stress,mobile phone,suicidal").

IEEE Xplore:

https://ieeexplore.ieee.org/search/searchresult.jsp?download-format=download-csv&bulkSetSize=2000&ranges%3D2007_2016_p_Publication_Year%26matchBoolean%3Dtrue%26searchField%3DSearch_All%26queryText%3D((smartphone%20OR%20mobile%20OR%20wearable%20OR%20.QT.smart%20phone.QT.%20OR%20.QT.app.QT.%20OR%20.QT.apps.QT.)%20AND%20(depression%20OR%20bipolar%20OR%20unipolar%20OR%20.QT.affective%20disorder.QT.%20or%20.QT.mental%20health.QT.%20or%20.QT.mood%20disorder.QT.)),2016%2F11%2F25

HTA/DARE from CRD database:

Results for: (smartphone OR mobile OR wearable OR smart phone OR app OR apps) AND (depression OR bipolar OR unipolar or affective disorder or mental health or mood disorder) FROM 2007 TO 2016
