# Supplementary material for: Correlations Between Objective Behavioral Features Collected From Mobile and Wearable Devices and Depressive Mood Symptoms in Patients With Affective Disorders: Systematic Review
Source: JMIR Mhealth Uhealth. 2018 Aug 13;6(8):e165. doi: 10.2196/mhealth.9691 (PMC6111148; doi:10.2196/mhealth.9691)
Supplement: Multimedia Appendix 2 [file mhealth_v6i8e165_app2.pdf]

Table S1. Study characteristics of studies with non-clinical samples. N/A: Not Available; N: Number of subjects; D: study Duration; F: Female; BDI: Becks Depression Inventory; DASS: Depression Anxiety Stress Scales; CDI: Children's Depression Inventory; PHQ: Patient Health Questionnaire; CES-D: Center for Epidemiological Studies Depression; SWLS: Satisfaction With Life Scale; QOL: Quality of Life; rPOMS: reduced Profile of Mood States; BRUMS: Brunel Mood Scale; PF: Penalized functional; P corr: Pearson correlation; SVM: Support Vector Machine; LME: Linear Mixed Effects; BHR: Bayesian hierarchical regression; GD: Group difference

**Main Author;year**	**Device;name**	**Recruitment**	**N (% F)**	**Age (m ± SD)**	**D (days)**	**Outcome**	**Method**
Asselbergs [3];2016	Android;funf	Flyers at Vrije Universiteit Amsterdam, The Nederlands	27 (78%)	21.1 ± 2.2	≈36	10p mood scale	P corr
Baras [4];2016	Android;Emotionstore	Students at the University of Madeira Funchal, Portugal	10 (10%)	N/A	14	Depression subscale of BRUMS	P corr
Becker [5];2016	Android;funf	Flyers at Vrije Universiteit Amsterdam, The Nederlands	27	Students	42	Mood	BHR
Ben-Zeev [17];2015	Android	Class announcement at the university.	47 (N/A)	22.5	70	PHQ-9	PF regression
Berke [8];2011	Mobile Sensing Platform device	Retirement community	8 (50%)	85.3 ± 4.1	10	CES-D	P corr
Canzian [9];2015	Android;MoodTraces	Academic mailing list + social media	28 (46%)	31	71	PHQ-8	P corr
Cho [1]; 2016	Phone	Korea University Ansan Hospital	532 (56%)	57	N/A	BDI	P corr
Chow [10];2017	Android	Advertisement through university mail	72 (51%)	19.8 ± 2.4	17	DASS-21	GME model
DeMasi [11];2016	Android	University of California, Berkeley USA	44 (61%)	N/A	56	BDI	Linear regression
Edwards [12];2016	Digi-Walker Pedometer	Word of mouth to students	39 (59%)	21.82	7	PHQ-9	Low activity vs Control
Farhan [13];2016	Android & iOS;LifeRhythm	e-mail + flyers, University of Connecticut, USA	79 (74%)	18-25	N/A	PHQ-9	P corr
Mark [19];2016	Fitbit Flex	Research division in U.S.	40 (50%)	N/A	12	Affect balance	LME
Matic [16]; 2011	Windows M. 6.5;MyExperience	Local research center	9 (33%)	28.4 ± 2.8	7	rPOMS	Spearmans corr
Mehrotra	Android	N/A	25 (N/A)	N/A	30	PHQ-8	Kendall’s

[18];2016							rank corr
Mestry [2]; 2015	Android 2.3+	Sardar Patel Institute of Technology Mumbai, India	2 (50%)	22	≈34	DASS21	P corr
Pillai [20]; 2014	Actigraph	Student enrolled from psychology courses	39 (74%)	19.55 ± 3.2	7	BDI	P corr
Saeb [6];2015	Android;Purple Robot	Craigslist advertisement	28 (71%)	28.9 ± 10.1	14	PHQ-9	P corr
Saeb [7];2016	Android	Studentlife data	48 (21%)	Students	70	PHQ-9	P corr
Wang [14];2015	Android;Studentlife	Students at Dartmouth College, USA	37 (N/A)	N/A	70	PHQ-9	P corr
Wang [15];2014	Android;Studentlife	Students at Dartmouth College, USA	48 (21%)	N/A	70	PHQ-9	P corr (post)

Table S2. Study findings for studies with nonclinical samples. F: Female; M: Male; pat: patient; contr: healthy control; *P < .05; **P < .01; °: Significance not reported; ¥: Human labeled, not objective

Main Author;year	Feature category	Sensor	Feature;description	Results on outcome
Asselbergs [3];2016	Physical activity	Accelerometer	Vigorous activity	r = .116**
	Device	Screen	Screen active frequency	r = .034
	Device	Screen	Screen active duration	r = -.074*
	Device	Phone	Images taken	r = .062
Baras [4];2016	Social	SMS log	SMS recieved	r = .542°
	Social	SMS log	SMS sent	r = .573°
Becker [5];2016	Physical activity	Accelerometer	Vigorous activity	β = 0.08
Ben-Zeev [17];2015	Social	Microphone	Speak duration	*P* = 0.048*
	Location	GPS+WIFI	Geospatial; log-transformed total distance	*P* = 0.022*
	Physical activity	Accelerometer	Kinesthetic; number of active periods	*P* > 0.05
	Subject	Multi sensor	Sleep duration; measured with screen, accelerometer, sound and light	*P* = 0.028*
Berke [8];2011	Social	Microphone	Speaking duration	r = -.73
Canzian [9];2015	Location	GPS	DT; Total distance covered	r = -.0252
	Location	GPS	DM; Maximum distance between two location clusters	r = -.0161
	Location	GPS	Coverage area; distances from locations to center	r = -.0090
	Location	GPS	Standard deviation of displacement	r = .0210
	Location	GPS	DH; Maximum distance between home and location	r = -.0175
	Location	GPS	Ndif; The number of different places visited	r = -.0203
	Location	GPS	Routine index; difference between daily mobility behavior	r = .0359
Cho [1]; 2016	Social	Call log	Call frequency	F/M: r = 0.049/-0.052
	Social	Call log	Call duration	F/M: r = 0.059/-0.031
Chow [10];2017	Location	GPS	Home stay (10AM-6PM)	Std. β = 0.03
DeMasi [11];2016	Subject	Accelerometer	STD of sleep	β = 7.2**
	Physical activity	Accelerometer	Activity (daytime)	β ≈ 0
	Physical activity	Accelerometer	STD stillness activity	β = -3.3**
	Physical activity	Accelerometer	Entropy	β ≈ 0

Edwards [12];2016	Physical activation	Pedometer	Activity (Step counter)	F = 11.85**
Farhan [13];2016	Location	GPS	Location variance; Variability in a subjects GPS location	r = -.15
	Location	GPS	Normalized distance; The amount of movement normalize with time period	r = -.13
	Physical activity	GPS	Movement speed	r = -.09
	Physical activity	GPS	Activity (duration)	r = .06
	Location	GPS	Entropy; The variability of the time the participant spent at the location clusters	r = -.16*
	Location	GPS	Normalized Entropy	r = -.21**
	Location	GPS	Home stay	r = .18*
	Location	GPS	Number of clusters	r = -.09
	Physical activity	Accelerometer	Activity	r = -.11
	Physical activity	Accelerometer	Inactive	r = .10
Mark [19];2016	Subject	Accelerometer	Sleep duration	β = 0.02**
	Physical activity	Accelerometer	Activity	β = 0.0001
Matic [16]; 2011	Physical activity	Accelerometer	Activity	r = -.26*
	Location	FM-position	Breaks; time spent in break room or balcony	r = -.21
Mehrotra [18];2016	Device	Notification	Acceptance; % notifications clicked	|r| < .2**
	Device	Notification	ST; time from arrives until seen	|r| < .2
	Device	Notification	DT; time from seen until acted upon	|r| < .2
	Device	Notification	RT; Response time (ST + DT)	|r| < .2**
	Device	App	App frequency (number of lunched apps)	|r| < .2
	Device	App	App duration	|r| < .2
	Device	Screen	Screen active duration	|r| < .2**
	Device	Screen	Screen active frequency	|r| < .2
	Device	Screen	Screen unlocks	|r| < .2**
Mestry [2]; 2015	Device	App	Communication apps used	r = -.33**
	Location	Internet	Distinct location visited – found through Telephony Manager	r = .09
	Device	Data usage	Network data transmitted	r = .07
	Social	Call log	Call duration (incoming)	r = .22
	Device	Screen	Screen active duration (between 9pm-10pm)	r = -.03
Pillai [20]; 2014	Subject	Accelerometer	Sleep onset latency	r = .21*
	Subject	Accelerometer	Sleep duration	r = .05

	Subject	Accelerometer	Sleep efficiency	r = -.15*
Saeb [6];2015	Location	GPS	Entropy	r = -.42
	Location	GPS	Normalized entropy	r = -.58*
	Location	GPS	Location variance	r = -.58*
	Location	GPS	Home stay	r = .49*
	Location	GPS	Transition time	r = .21
	Location	GPS	Circadian rhythm	r = -.63**
	Location	GPS	Number of clusters	r = -.09
	Physical activity	GPS	Activity	r = -.08
	Device	Screen	Screen active duration	r = .54*
	Device	Screen	Screen active frequency	r = .52*
Saeb [7];2016	Location	GPS	Location variance	r = -.43*
	Location	GPS	Circadian movement	r = -.48*
	Location	GPS	Speed mean	r = -.06
	Location	GPS	Speed variance	r = -.06
	Location	GPS	Number of clusters	r = -.44*
	Location	GPS	Entropy	r = -.46*
	Location	GPS	Normalized entropy	r = -.44*
	Location	GPS	Raw entropy	r = .22
	Location	GPS	Home stay	r = .43*
	Location	GPS	Transition time	r = -.32
	Physical	GPS	Total distance	r = -.18
Wang [14];2015	Subject	Camera	Image brightness, contrast and saturation	All three not significant
	Subject	¥ Camera	Label: laying down; if person, during EMA, was laying down	r = .51**
Wang [15];2014	Subject	Multiple-sensor	Sleep duration; light, lock-state, accelerometer and microphone	r = -.382*
	Social	Call log	Call frequency	r = -.387*
	Social	Call log	Call frequency (evening)	r = -.345*
	Social	Call log	Call duration	r = -.328*
	Location	Bluetooth	Co-located Bluetooth deices	r = -.362*

1. Cho YM, Lim HJ, Jang H, Kim K, Choi JW, Shin C, Lee SK, Kwon JH, Kim N. A cross-sectional study of the association between mobile phone use and symptoms of ill health. 2016;1–7.
2. Mestry M, Mehta J, Mishra A, Gawande K. Identifying associations between smartphone usage and mental health during depression, anxiety and stress. Proc - 2015 Int Conf Commun Inf Comput Technol ICCICT 2015 2015; [doi: 10.1109/ICCICT.2015.7045656]
3. Asselbergs J, Ruwaard J, Ejdys M, Schrader N, Sijbrandij M, Riper H. Mobile Phone-Based Unobtrusive Ecological Momentary Assessment of Day-to-Day Mood: An Explorative Study. J Med Internet Res [Internet] 2016;18(3):e72. PMID:27025287
4. Baras K, Soares L, Paulo N, Barros R. “Smartphine”: Supporting students’ well-being according to their calendar and mood. 2016 Int Multidiscip Conf Comput Energy Sci Split 2016 2016; [doi: 10.1109/SpliTech.2016.7555919]
5. Becker D, Bremer V, Funk B, Asselbergs J, Riper H, Ruwaard J. How to Predict Mood? Delving into Features of Smartphone-Based Data. Twenty-second Am Conf Inf Syst 2016;1–10.
6. Saeb S, Zhang M, Karr CJ, Schueller SM, Corden ME, Kording KP, Mohr DC. Mobile phone sensor correlates of depressive symptom severity in daily-life behavior: An exploratory study. J Med Internet Res 2015;17(7):1–11. PMID:26180009
7. Saeb S, Lattie EG, Schueller SM, Kording KP, Mohr DC. The relationship between mobile phone location sensor data and depressive symptom severity. PeerJ [Internet] 2016;4:e2537. PMID:28344895
8. Berke EM, Choudhury T, Ali S, Rabbi M. Objective measurement of sociability and activity: Mobile sensing in the community. Ann Fam Med 2011;9(4):344–350. PMID:21747106
9. Canzian L, Musolesi M. Trajectories of Depression : Unobtrusive Monitoring of Depressive States by means of Smartphone Mobility Traces Analysis. Proc 2015 ACM Int Jt Conf Pervasive Ubiquitous Comput 2015;1293–1304. [doi: 10.1145/2750858.2805845]
10. Chow PI, Fua K, Huang Y, Bonelli W, Xiong H, Barnes LE, Teachman BA. Using Mobile Sensing to Test Clinical Models of Depression, Social Anxiety, State Affect, and Social Isolation Among College Students. J Med Internet Res [Internet] 2017;19(3):e62. PMID:28258049
11. Demasi O, Aguilera A, Recht B. Detecting Change in Depressive Symptoms from Daily Wellbeing Questions , Personality , and Activity. 2016;22–29.
12. Edwards MK, Loprinzi PD. Effects of a Sedentary Behavior–Inducing Randomized Controlled Intervention on Depression and Mood Profile in Active Young Adults. Mayo Clin Proc [Internet] 2016 [cited 2017 May 11];91(8):984–998. PMID:27492908
13. Farhan AA, Yue C, Morillo R, Ware S, Lu J, Bi J, Kamath J, Russell A, Bamis A, Wang B. Behavior vs . Introspection : Refining prediction of clinical depression via smartphone sensing data. 2016;30–37.
14. Wang R, Campbell AT, Zhou X. Using opportunistic face logging from smartphone to infer mental health. Proc 2015 ACM Int Jt Conf Pervasive Ubiquitous Comput Proc 2015 ACM Int Symp Wearable Comput - UbiComp ’15 [Internet] 2015;683–692. [doi: 10.1145/2800835.2804391]
15. Wang R, Chen F, Chen Z, Li T, Harari G, Tignor S, Zhou X, Ben-Zeev D, Campbell AT. StudentLife: assessing mental health, academic performance and behavioral trends of college students using smartphones. Proc 2014 ACM Int Jt Conf Pervasive Ubiquitous Comput 2014;3–14. [doi: 10.1145/2632048.2632054]
16. Matic A, Osmani V, Popleteev A, Mayora-Ibarra O. Smart phone sensing to examine effects of social interactions and non-sedentary work time on mood changes. Lect Notes Comput Sci (including Subser Lect Notes Artif Intell Lect Notes Bioinformatics) 2011;6967 LNAI:200–213. [doi: 10.1007/978-3-642-24279-3_21]
17. Ben-Zeev D, Scherer EA, Wang R, Xie H, Campbell AT. Next-Generation Psychiatric Assessment: Using Smartphone Sensors to Monitor Behavior

and Mental Health HHS Public Access. Psychiatr Rehabil J [Internet] 2015 [cited 2017 Feb 4];38(3):218–226. PMID:25844912
18. Mehrotra A, Hendley R, Musolesi M. Towards multi-modal anticipatory monitoring of depressive states through the analysis of human-smartphone interaction. Proc 2016 ACM Int Jt Conf Pervasive Ubiquitous Comput Adjun - UbiComp '16 [Internet] 2016 [cited 2017 Feb 5]. p. 1132–1138. [doi: 10.1145/2968219.2968299]
19. Mark G, Czerwinski M, Iqbal S, Johns P. Workplace Indicators of Mood: Behavioral and Cognitive Correlates of Mood Among Information Workers. Proc 6th Int Conf Digit Heal Conf [Internet] 2016;29–36. [doi: 10.1145/2896338.2896360]
20. Pillai V, Steenburg LA, Ciesla JA, Roth T, Drake CL. A seven day actigraphy-based study of rumination and sleep disturbance among young adults with depressive symptoms. J Psychosom Res [Internet] Elsevier Inc.; 2014;77(1):70–75. PMID:24913345
