# Supplementary material for: Correlations Between Objective Behavioral Features Collected From Mobile and Wearable Devices and Depressive Mood Symptoms in Patients With Affective Disorders: Systematic Review
Source: JMIR Mhealth Uhealth. 2018 Aug 13;6(8):e165. doi: 10.2196/mhealth.9691 (PMC6111148; doi:10.2196/mhealth.9691)
Supplement: Multimedia Appendix 4 [file mhealth_v6i8e165_app4.pdf]

Table S3. Study characteristics of studies with a medical diagnose of either Major Depressive Disorder (MDD) or Bipolar Disorder (BD). N/A: Not Available; N: Number of subjects;D: study Duration;F: Female; CES-D: Center for Epidemiological Studies Depression; HDRS: Hamilton Rating Scale for Depression; YRMS: Young Mania Rating Scale; P Corr: Pearson correlation; RF: Random forest; SRM: Social Rhythm Metric; SVM: Support Vector Machine; QIDS-SR: Quick Inventory of Depressive Symptomatology - Self Reported; QDA: quadratic discriminant analysis; GD: Group difference; QIDS-C: Inventory of Depressive Symptomatology - Clinician rated; ADL: Activities of Daily Living; RE; Random effects; IDS-C: inventory for depressive symptomatology, Clinicial-Rated; MLR: Multivariable logistic regression.

**Main Author;year**	**Device;name**	**Recruitment**	**N (% F)**	**Age (m ± SD)**	**D (days)**	**Outcome**	**Method**
Abdullah [1];2016	Android;MoodRhythm	Western Psychiatric Institute and clinic	7 (71%)	25-64	28	SRM II-5	SVM
Alvarez-Lozano [2];2014	Android;MONARCA	Cl. For Affective Disorders, Psych. Center Copenhagen, Denmark	18 (N/A)	N/A	150	-3:3 Mood scale	P corr
Beiwinkel [3];2016	Android:SIMBA	Psychiatric clinic, Lower Saxon Germany	13 (39%)	47.2 ± 3.8	365	HDRS	RE model
Berle [4]; 2010	Actigraph	N/A	23 (54%)	42.8 ± 11	14	GD	Univariate t-test
Dickerson [5]; 2011	iOS;Empath	Volunteer	1 (100%)	83	14	10p Mood scale	Linear regression
Doryab [6];2014	Anroid	Mental health clinic	6 (50%)	>18	≈20	CES-D	P corr
Faurholt-Jepsen [7];2015	Android;MONARCA	Cl. For Affective Disorders, Psych. Center Copenhagen, Denmark	61 (67%)	29.3 ± 8.4	N/A	HDRS-17	LME model
Faurholt-Jepsen [8];2014	Android;MONARCA	Cl. For Affective Disorders, Psych. Center Copenhagen, Denmark	17 (71%)	33.4 ± 9.5	90	HDRS-17	LME model
Faurholt-Jepsen [9];2016	Android;MONARCA	Cl. For Affective Disorders, Psych. Center Copenhagen, Denmark	28 (68%)	30.3 ± 9.3	84	Mood scale	RF classification
Faurholt-Jepsen [10];2012	Actiheart	Cl. For Affective Disorders, Psych. Center Copenhagen, Denmark	20 (60%)	45.2 ± 12	3	GD	Univariate t-test
Faurholt-Jepsen [11];2015	Actiheart	Cl. For Affective Disorders, Psych. Center Copenhagen, Denmark	18 (61%)	45.6 ± 11.1	3	HDRS-17	Linear regression
Faurholt-Jepsen [12];2016	Android;MONARCA	Cl. For Affective Disorders, Psych. Center Copenhagen, Denmark	29 (62%)	30.2 ± 8.8	84	HDRS-17	LME
Gershon [13];2016	Actigraph	Online advertisements + flyers posted in the community around San Francisco	37 (62%)	34.4 ± 10.4	46	GD	Univariate t-test

Gonzalez; [14];2014	Actigraph	County and community hospitals, university medical center etc throughout Dallas County	42 (64%)	41.0 ± 11.2	7	IDS-C-30	P corr
Grünerbl [15]; 2015	Android	Rural area psychiatric hospital Austria	10 (80%)	33-48	84	-3:3 Mood scale	NBC
Guidi [16]; 2015	Android	Department of psychiatry and mental health, Strasbourg University hospital	1 (100%)	36	98	Mood state	Spearman corr
Hauge [17]; 2011	Actigraph	University Hospital of Bergen	25 (44%)	42.9 ± 10.7	14	GD	Univariate t-test
Krane-Gartiser [18]; 2014	Actigraph	inpatients at the Department of Psychiatry, St. Olav's University Hospital, Trondheim, Norway	12 (58%)	39.9 ± 15.6	1	GD	Univariate t-test
Loprinzi [19]; 2014	Actigraph	NHANES data	2574 (51%)	46.3	7	GD	MLR
Miwa [20]; 2017	Armband;SenseWear Pro	N/A	5 (0%)	35.1	87	GD	t-test
Muaremi [21];2014	Android	Psychiatric hospital Hall, Tirol Austria	6 (N/A)	18-65	≈76	-3:3 Mood scale	RF classification
O'Brien [22]; 2016	Actigraph	Care services across North Easts of England	59 (73%)	74 ± 6	7	GD & MADRS	t-test & P corr
Osmani [23];2013	Android	N/A	5 (100%)	N/A	90	Mental health state	P corr
Palmius [24]; 2016	Android;AMoSS	Oxford area	36 (75%)	44 ± 14	60	QIDS-SR16	QDA (GD)
St-Amand [25];2013	Actigraph	mood disorders' clinic of a university affiliated mental health center	14 (50%)	44.6 ± 11	14	GD	Univariate t-test
Todder [26]; 2019	Actigraph	N/A	27 (48%)	49 ± 13	7	GD	Univariate t-test

1. Abdullah S, Matthews M, Frank E, Doherty G, Gay G, Choudhury T. Automatic detection of social rhythms in bipolar disorder. J Am Med Informatics Assoc 2016;23(3):538–543. PMID:26977102
2. Alvarez-lozano J, Frost M, Osmani V, Bardram J, Kessing LV, Mayora O, Faurholt-Jepsen M. Tell me your apps and I will tell you your mood: correlation of apps usage with bipolar disorder state. 7th Int Conf Pervasive Technol Relat to Assist Envotinments 2014;1(212). [doi: 10.1145/2674396.2674408]
3. Beiwinkel T, Kindermann S, Maier A, Kerl C, Moock J, Barbian G, Rössler W. Using Smartphones to Monitor Bipolar Disorder Symptoms: A Pilot Study. JMIR Ment Heal [Internet] 2016;3(1):e2. PMID:26740354
4. Berle JO, Hauge ER, Oedegaard KJ, Holsten F, Fasmer OB. Actigraphic registration of motor activity reveals a more structured behavioural pattern in schizophrenia than in major depression. BMC Res Notes 2010;3. PMID:20507606
5. Dickerson RF, Gorlin EI, Stankovic JA. Empath: a continuous remote emotional health monitoring system for depressive illness. Proc 2nd Conf Wirel Heal - WH '11 [Internet] 2011;Art. 5. [doi: 10.1145/2077546.2077552]
6. Doryab A, Min JK, Wiese J, Zimmerman J, Hong JI. Detection of behavior change in people with depression. AAAI Work Work Twenty-Eighth AAAI Conf Artif Intell 2014;12–16.
7. Faurholt-Jepsen M, Vinberg M, Frost M, Christensen EM, Bardram JE, Kessing LV. Smartphone data as an electronic biomarker of illness activity in bipolar disorder. Bipolar Disord [Internet] 2015 [cited 2017 Feb 5];17(7):715–728. PMID:26395972
8. Faurholt-Jepsen M, Frost M, Vinberg M, Christensen EM, Bardram JE, Kessing LV. Smartphone data as objective measures of bipolar disorder symptoms. Psychiatry Res Elsevier; 2014;217(1):124–127.
9. Faurholt-Jepsen M, Busk J, Frost M, Vinberg M, Christensen EM, Winther O, Bardram JE, Kessing L V. Voice analysis as an objective state marker in bipolar disorder. Transl Psychiatry [Internet] Nature Publishing Group; 2016;6(7):e856. PMID:27434490
10. Faurholt-jepsen M, Brage S, Vinberg M, Margrethe E, Knorr U, Mørch H, Vedel L. Differences in psychomotor activity in patients suffering from unipolar and bipolar affective disorder in the remitted or mild / moderate depressive state. J Affect Disord [Internet] Elsevier B.V.; 2012;141(2–3):457–463. [doi: 10.1016/j.jad.2012.02.020]
11. Faurholt-Jepsen M, Brage S, Vinberg M, Jensen HM, Christensen EM, Knorr U, Kessing LV. Electronic monitoring of psychomotor activity as a supplementary objective measure of depression severity. Nord J Psychiatry 2015;69(2):118–125. PMID:25131795
12. Faurholt-Jepsen M, Vinberg M, Frost M, Debel S, Margrethe Christensen E, Bardram JE, Kessing LV. Behavioral activities collected through smartphones and the association with illness activity in bipolar disorder. Int J Methods Psychiatr Res 2016; PMID:21516187
13. Gershon A, Ram N, Johnson SL, Harvey AG, Zeitzer JM. Daily actigraphy profiles distinguish depressive and interepisode states in bipolar disorder. Clin Psychol Sci 2016;4(4):641–650. PMID:24655651
14. Gonzalez R, Tamminga CA, Tohen M, Suppes T. The relationship between affective state and the rhythmicity of activity in bipolar disorder. J Clin Psychiatry 2014;75(4):1–14. PMID:24500063
15. Grünerbl A, Muaremi A, Osmani V, Bahle G, Öhler S, Tröster G, Mayora O, Haring C, Lukowicz P. Smart-Phone Based Recognition of States and State Changes in Bipolar Disorder Patients. IEEE J Biomed Heal Informatics [Internet] 2015;19(1):140–148. PMID:25073181
16. Guidi A, Vanello N, Bertschy G, Gentili C, Landini L, Scilingo EP. Automatic analysis of speech F0 contour for the characterization of mood changes in bipolar patients. Biomed Signal Process Control [Internet] 2015 [cited 2017 Feb 4];17:29–37. [doi: 10.1016/j.bspc.2014.10.011]
17. Hauge ER, Berle JØ, Oedegaard KJ, Holsten F, Fasmer OB. Nonlinear analysis of motor activity shows differences between schizophrenia and

depression: A study using fourier analysis and sample entropy. PLoS One 2011;6(1):1–10. PMID:21297977
18. Krane-Gartiser K, Henriksen TEG, Morken G, Vaaler A, Fasmer OB. Actigraphic assessment of motor activity in acutely admitted inpatients with bipolar disorder. PLoS One 2014;9(2). PMID:24586883
19. Loprinzi PD, Mahoney S. Concurrent occurrence of multiple positive lifestyle behaviors and depression among adults in the United States. J Affect Disord [Internet] Elsevier; 2014;165:126–130. [doi: 10.1016/j.jad.2014.04.073]
20. Miwa H, Sasahara S, Matsui T. Roll-over detection and sleep quality measurement using a wearable sensor. Conf Proc IEEE Eng Med Biol Soc [Internet] IEEE; 2007 Aug [cited 2017 Feb 4];2007:1507–1510. PMID:18002253
21. Muaremi A, Gravenhorst F, Grünerbl A, Arnrich B, Tröster G. Assessing bipolar episodes using speech cues derived from phone calls. Lect Notes Inst Comput Sci Soc Telecommun Eng LNICST 2014. p. 103–114. [doi: 10.1007/978-3-319-11564-1_11]
22. O'Brien JT, Gallagher P, Stow D, Hammerla N, Ploetz T, Firbank M, Ladha C, Ladha K, Jackson D, McNaney R, Ferrier IN, Olivier P. A study of wrist-worn activity measurement as a potential real-world biomarker for late-life depression. Psychol Med [Internet] 2016;No-Specified. PMID:27667663
23. Osmani V, Maxhuni A, Grunerbl A, Lukowicz P, Haring C, Mayora O. Monitoring activity of patients with bipolar disorder using smart phones. Proc Int Conf Adv Mob Comput Multimed [Internet] 2013;85–92. [doi: 10.1145/2536853.2536882]
24. Palmius N, Tsanas A, Saunders KEA, Bilderbeck AC, Geddes JR, Goodwin GM, De Vos M. Detecting Bipolar Depression from Geographic Location Data. IEEE Trans Biomed Eng [Internet] 2016 [cited 2017 Feb 6];1–1. [doi: 10.1109/TBME.2016.2611862]
25. St-Amand J, Provencher MD, Bélanger L, Morin CM. Sleep disturbances in bipolar disorder during remission. J Affect Disord 2013;146(1):112–119. PMID:22884237
26. Todder D, Caliskan S, Baune BT. Longitudinal changes of day-time and night-time gross motor activity in clinical responders and non-responders of major depression. World J Biol Psychiatry 2009;10(4):276–284. PMID:19921969
