# Supplementary material for: Correlations Between Objective Behavioral Features Collected From Mobile and Wearable Devices and Depressive Mood Symptoms in Patients With Affective Disorders: Systematic Review
Source: JMIR Mhealth Uhealth. 2018 Aug 13;6(8):e165. doi: 10.2196/mhealth.9691 (PMC6111148; doi:10.2196/mhealth.9691)
Supplement: Multimedia Appendix 5 [file mhealth_v6i8e165_app5.pdf]

Table S4. Study findings for studies with a medical diagnose of either Unipolar Disorder (UD) or Bipolar Disorder (BD). F: Female; M: Male; pat: patient; contr: healthy control; *P < .05; **P < .01; °: Significance not reported; y: Human labeled, not objective

Main Author;year	Feature category	Sensor	Feature; description	Results on outcome
Abdullah [1];2016	Location	GPS	Total distance	F1 = 85.5%, w = 1.56*10^-2
	Location	GPS	Number of clusters;	F1 = 85.5%, w = 3.27*10^-3
	Physical activity	Accelerometer	Activity (duration)	F1 = 85.5%, w = -3.79*10^-3
	Social	Microphone	Call frequency	F1 = 85.5%, w = 7.69 * 10^-5
Alvarez-Lozano [2];2014	Device	App	Browser apps (duration)	r = .28*
	Device	App	Social apps (duration)	r = -.23*
	Device	App	Apps running during active screen (number)	r = .31*
	Device	Screen	Screen active duration (seconds)	r = -.38*
Beiwinkel [3];2016	Physical activity	GPS	Distance (km)	β = 0.03
	Location	GSM	Cell tower (ID numbers)	β = -0.11*
	Physical activity	Accelerometer	Activity (duration)	β = 0.02
	Social	Call log	Outgoing call frequency	β = -0.07
	Social	Call log	Outgoing call duration	β = -0.09
	Social	SMS log	Outgoing SMS frequency	β = -0.28**
Berle [4]; 2010	Physical activity	Accelerometer	Activity (duration)	187 patients vs 286 control**
	Physical activity	Accelerometer	Activity night (23-06)	61 patients vs 89
Dickerson [5]; 2011	Subject	Microphone	Fundamental freq; First harmonic in frequency decomposite of the voice signal	β = -0.01
	Subject	Microphone	Speech pause time; within the morning and evening voice session, amount of time with no sound detection	β = -0.34**
Doryab [6];2014	Location	GPS	Home stay	F: p = .5*
	Environment	Internet	Humidity	F / M: p = -.4 / -.5
	Social	Call log	Call frequency	M: p = -.5
Faurholt-Jepsen [7];2015	Social	Call log	Call frequency (incoming)	β = 0.024
	Social	Call log	Call duration (incoming)	β = 17.15*
	Social	SMS log	SMS frequency (incoming)	β = -0.029
	Social	SMS log	SMS frequency (outgoing)	β = 0.022
	Social	Call log	Call frequency (outgoing)	β = 0.030
	Social	Call log	Call duration (outgoing)	β = 26.33*
Faurholt-Jepsen [8];2014	Location	GSM	Cell tower	β = -0.43
	Device	Screen	Screen active duration	β = 16.38

	Social	Call log	Call frequency (outgoing)	β = -0.020
	Social	SMS log	SMS frequency (outgoing)	β = -0.017
Faurholt-Jepsen [9];2016	Subject	Microphone	Voice features; 6552 features from openSMILE	Dep vs euth: SE/SP = 81%/56%, Acc = 68%
	Device	Multi sensor	Voice + objective features: Microphone, SMS, phone logs, screen	Dep vs euth: SE/SP = 78%/47%, Acc = 62%
Faurholt-Jepsen [10];2012	Bio	ECG	Heart rate (sleep)	63.3 patient vs 54.9 control**
	Physical activity	Accelerometer	Activity	0.085 pat vs 0.11 contr
	Physical activity	ECG	Fitness	34 pat vs 42.2 contr*
	Physical activity	ECG + Accelerometer	Activity energy expenditure	35 vs 52*
Faurholt-Jepsen [11];2015	Bio	ECG	Heart rate (sleep)	β = 0.40
	Physical activity	ECG	Fitness	β = -0.57
	Physical activity	ECG + Accelerometer	Activity energy expenditure	β = -0.64
Faurholt-Jepsen [12];2016	Social	SMS log	SMS frequency (incoming)	β = -0.20
	Social	SMS log	SMS frequency (outgoing)	β = 0.07
	Physical activity	Screen	Screen active duration	β = 209.6*
	Physical activity	Screen	Screen active frequency	β = -0.13
	Social	Call log	Call duration	β = 0.66
	Location	GSM	Cell tower	β = -0.56**
	Social	SMS log	SMS characters (incoming)	β = 1.62
	Social	SMS log	SMS characters (outgoing)	β = 1.60
	Social	Call log	Call frequency (incoming)	β = 0.05*
	Social	Call log	Call frequency (outgoing)	β = -0.12*
	Social	Call log	Call missed	β = 0.05**
	Social	Call log	Call duration (incoming)	β = 21.5

	Social	Call log	Call duration (outgoing)	β = -1.8
Gershon [13];2016	Physical activity	Accelerometer	Activity	fPCA1 = -849.49*
Gonzalez; [14];2014	Physical activity	Accelerometer	Autocorrelation; rhythmicity	r = -.119
	Physical activity	Accelerometer	Circadian quotient; robustness of rhythm	r = .0083
Grünerbl [15]; 2015	Physical activity	Accelerometer	Multiple features	Recall/precision =62.9%/64.8%, Acc = 71.7%
	Location	GPS	Multiple features	Recall/precision =72.3%/76.5%, Acc = 81.7%
	Social	Call log	Multiple features	Recall/precision = 54.4%/37.3, Acc = 64.2%
	Subject	Microphone	Multiple features	Recall/precision = 61.3%/24.2%, Acc = 69.3%
Guidi [16]; 2015	Subject	Microphone	MAD_meanF0; absolute deviation of median vocal fundamental frequency F0	r = .54*
Hauge [17]; 2011	Physical activity	Accelerometer	Sample entropy (m = 2, r = 0.2); the degree of regularity	0.62 pat vs 0.59 contr
	Physical activity	Accelerometer	Activity	11057 pat vs 17031 contr **
	Physical activity	Accelerometer	RMSSD; difference in successive counts from minute to minute	95.5 pat vs 86.1 contr
	Physical activity	Accelerometer	Standard deviation of activity	106.2 pat vs 90.8 contr **
	Physical activity	Accelerometer	Fourier analysis; ratios between variance in the low frequency and the high frequency parts of the spectrum	0.46 pat vs 0.32 contr *
Krane-Gartiser [18]; 2014	Physical activity	Accelerometer	Activity	128 vs 203 *
	Physical activity	Accelerometer	Standard deviation of activity	179.4 vs 147.3
	Physical activity	Accelerometer	RMSSD	150.5 vs 99.1 *
Loprinzi [19]; 2014	Physical activity	Accelerometer	MVPA; minutes with > 2020 activity counts. The risk of having depression	Odds ration = 0.46*
Miwa [20]; 2017	Subject	Accelerometer	Sleep duration	μ UD/NC: 399/369 **
	Subject	Accelerometer	Quality of sleep (Deep sleep/total sleep)	μ UD/NC: 0.43/0.58 **
Muaremi [21];2014	Social	Microphone	Speak duration	F1 = 82% best feature
	Social	Microphone	Call duration maximum	F1 = 82% 5th feature
	Social	Microphone	Harmonics-to-noise ration	F1 = 82% 2nd feature
	Social	Microphone	Short turns/utterances during conversations	F1 = 82% 3rd feature
	Social	Microphone	Standard deviation of the pitch frequencfy	F1 = 82% 4rd feature
O'Brien [22];	Physical activity	Accelerometer	Activity	μ LLD/NC 0.17/0.20, t =

				3.69**
2016	Physical activity	Accelerometer	Jerk; a measure of quick movements	µ LLD/NC 0.001/0.001, t = 4.06**
	Physical activity	Accelerometer	Entropy-movement; Higher: less predictability and less repetitive movements	µ LLD/NC 2.50/2.81, t = 3.56**
	Physical activity	Accelerometer	Entropy movement between 06:12	r = -0.37*
Osmani [23];2013	Physical activity	Accelerometer	Activity (morning: 6AM-12PM)	r = 0.581*
	Physical activity	Accelerometer	Activity (afternoon: 12PM-06PM)	r = -0.542*
	Physical activity	Accelerometer	Activity (Evening: 06PM-12AM)	r = 0.619*
Palmius [24]; 2016	Location	Multi sensor	50 features from GPS, WIFI, AP, GSM	F1 = 85.5%, Acc = 0.849
St-Amand [25];2013	Physical activity	Accelerometer	Activity	160.7 pat vs 254.5 contr **
	Subject	Accelerometer	Sleep onset latency	14.6 pat vs 15.7 contr
	Subject	Accelerometer	Wake after sleep onset	53.7 pat vs 53.9 contr
	Subject	Accelerometer	Sleep duration	416.8 pat vs 416 contr
	Subject	Accelerometer	Sleep efficiency	85.4 pat vs 85.8 contr
Todder [26]; 2019	Physical activity	Accelerometer	Activity (day: 8-20)	161 pat vs 210 contr **
	Physical activity	Accelerometer	Activity (night: 00-06)	85.2 pat vs 121.4 contr **

1. Abdullah S, Matthews M, Frank E, Doherty G, Gay G, Choudhury T. Automatic detection of social rhythms in bipolar disorder. J Am Med Informatics Assoc 2016;23(3):538–543. PMID:26977102
2. Alvarez-lozano J, Frost M, Osmani V, Bardram J, Kessing LV, Mayora O, Faurholt-Jepsen M. Tell me your apps and I will tell you your mood: correlation of apps usage with bipolar disorder state. 7th Int Conf Pervasive Technol Relat to Assist Envotinments 2014;1(212). [doi: 10.1145/2674396.2674408]
3. Beiwinkel T, Kindermann S, Maier A, Kerl C, Moock J, Barbian G, Rössler W. Using Smartphones to Monitor Bipolar Disorder Symptoms: A Pilot Study. JMIR Ment Heal [Internet] 2016;3(1):e2. PMID:26740354
4. Berle JO, Hauge ER, Oedegaard KJ, Holsten F, Fasmer OB. Actigraphic registration of motor activity reveals a more structured behavioural pattern in schizophrenia than in major depression. BMC Res Notes 2010;3. PMID:20507606
5. Dickerson RF, Gorlin EI, Stankovic JA. Empath: a continuous remote emotional health monitoring system for depressive illness. Proc 2nd Conf Wirel Heal - WH '11 [Internet] 2011;Art. 5. [doi: 10.1145/2077546.2077552]
6. Doryab A, Min JK, Wiese J, Zimmerman J, Hong JI. Detection of behavior change in people with depression. AAAI Work Work Twenty-Eighth AAAI Conf Artif Intell 2014;12–16.
7. Faurholt-Jepsen M, Vinberg M, Frost M, Christensen EM, Bardram JE, Kessing LV. Smartphone data as an electronic biomarker of illness activity in bipolar disorder. Bipolar Disord [Internet] 2015 [cited 2017 Feb 5];17(7):715–728. PMID:26395972
8. Faurholt-Jepsen M, Frost M, Vinberg M, Christensen EM, Bardram JE, Kessing LV. Smartphone data as objective measures of bipolar disorder symptoms. Psychiatry Res Elsevier; 2014;217(1):124–127.
9. Faurholt-Jepsen M, Busk J, Frost M, Vinberg M, Christensen EM, Winther O, Bardram JE, Kessing L V. Voice analysis as an objective state marker in bipolar disorder. Transl Psychiatry [Internet] Nature Publishing Group; 2016;6(7):e856. PMID:27434490
10. Faurholt-jepsen M, Brage S, Vinberg M, Margrethe E, Knorr U, Mørch H, Vedel L. Differences in psychomotor activity in patients suffering from unipolar and bipolar affective disorder in the remitted or mild / moderate depressive state. J Affect Disord [Internet] Elsevier B.V.; 2012;141(2–3):457–463. [doi: 10.1016/j.jad.2012.02.020]
11. Faurholt-Jepsen M, Brage S, Vinberg M, Jensen HM, Christensen EM, Knorr U, Kessing LV. Electronic monitoring of psychomotor activity as a supplementary objective measure of depression severity. Nord J Psychiatry 2015;69(2):118–125. PMID:25131795
12. Faurholt-Jepsen M, Vinberg M, Frost M, Debel S, Margrethe Christensen E, Bardram JE, Kessing LV. Behavioral activities collected through smartphones and the association with illness activity in bipolar disorder. Int J Methods Psychiatr Res 2016; PMID:21516187
13. Gershon A, Ram N, Johnson SL, Harvey AG, Zeitzer JM. Daily actigraphy profiles distinguish depressive and interepisode states in bipolar disorder. Clin Psychol Sci 2016;4(4):641–650. PMID:24655651
14. Gonzalez R, Tamminga CA, Tohen M, Suppes T. The relationship between affective state and the rhythmicity of activity in bipolar disorder. J Clin Psychiatry 2014;75(4):1–14. PMID:24500063
15. Grünerbl A, Muaremi A, Osmani V, Bahle G, Öhler S, Tröster G, Mayora O, Haring C, Lukowicz P. Smart-Phone Based Recognition of States and State Changes in Bipolar Disorder Patients. IEEE J Biomed Heal Informatics [Internet] 2015;19(1):140–148. PMID:25073181
16. Guidi A, Vanello N, Bertschy G, Gentili C, Landini L, Scilingo EP. Automatic analysis of speech F0 contour for the characterization of mood changes in bipolar patients. Biomed Signal Process Control [Internet] 2015 [cited 2017 Feb 4];17:29–37. [doi: 10.1016/j.bspc.2014.10.011]
17. Hauge ER, Berle JØ, Oedegaard KJ, Holsten F, Fasmer OB. Nonlinear analysis of motor activity shows differences between schizophrenia and

depression: A study using fourier analysis and sample entropy. PLoS One 2011;6(1):1–10. PMID:21297977

18. Krane-Gartiser K, Henriksen TEG, Morken G, Vaaler A, Fasmer OB. Actigraphic assessment of motor activity in acutely admitted inpatients with bipolar disorder. PLoS One 2014;9(2). PMID:24586883
19. Loprinzi PD, Mahoney S. Concurrent occurrence of multiple positive lifestyle behaviors and depression among adults in the United States. J Affect Disord [Internet] Elsevier; 2014;165:126–130. [doi: 10.1016/j.jad.2014.04.073]
20. Miwa H, Sasahara S, Matsui T. Roll-over detection and sleep quality measurement using a wearable sensor. Conf Proc IEEE Eng Med Biol Soc [Internet] IEEE; 2007 Aug [cited 2017 Feb 4];2007:1507–1510. PMID:18002253
21. Muaremi A, Gravenhorst F, Grünerbl A, Arnrich B, Tröster G. Assessing bipolar episodes using speech cues derived from phone calls. Lect Notes Inst Comput Sci Soc Telecommun Eng LNICST 2014. p. 103–114. [doi: 10.1007/978-3-319-11564-1_11]
22. O'Brien JT, Gallagher P, Stow D, Hammerla N, Ploetz T, Firbank M, Ladha C, Ladha K, Jackson D, McNaney R, Ferrier IN, Olivier P. A study of wrist-worn activity measurement as a potential real-world biomarker for late-life depression. Psychol Med [Internet] 2016;No-Specified. PMID:27667663
23. Osmani V, Maxhuni A, Grunerbl A, Lukowicz P, Haring C, Mayora O. Monitoring activity of patients with bipolar disorder using smart phones. Proc Int Conf Adv Mob Comput Multimed [Internet] 2013;85–92. [doi: 10.1145/2536853.2536882]
24. Palmius N, Tsanas A, Saunders KEA, Bilderbeck AC, Geddes JR, Goodwin GM, De Vos M. Detecting Bipolar Depression from Geographic Location Data. IEEE Trans Biomed Eng [Internet] 2016 [cited 2017 Feb 6];1–1. [doi: 10.1109/TBME.2016.2611862]
25. St-Amand J, Provencher MD, Bélanger L, Morin CM. Sleep disturbances in bipolar disorder during remission. J Affect Disord 2013;146(1):112–119. PMID:22884237
26. Todder D, Caliskan S, Baune BT. Longitudinal changes of day-time and night-time gross motor activity in clinical responders and non-responders of major depression. World J Biol Psychiatry 2009;10(4):276–284. PMID:19921969
