# Supplementary figures and images for: Correlations Between Objective Behavioral Features Collected From Mobile and Wearable Devices and Depressive Mood Symptoms in Patients With Affective Disorders: Systematic Review
Source: JMIR Mhealth Uhealth. 2018 Aug 13;6(8):e165. doi: 10.2196/mhealth.9691 (PMC6111148; doi:10.2196/mhealth.9691)

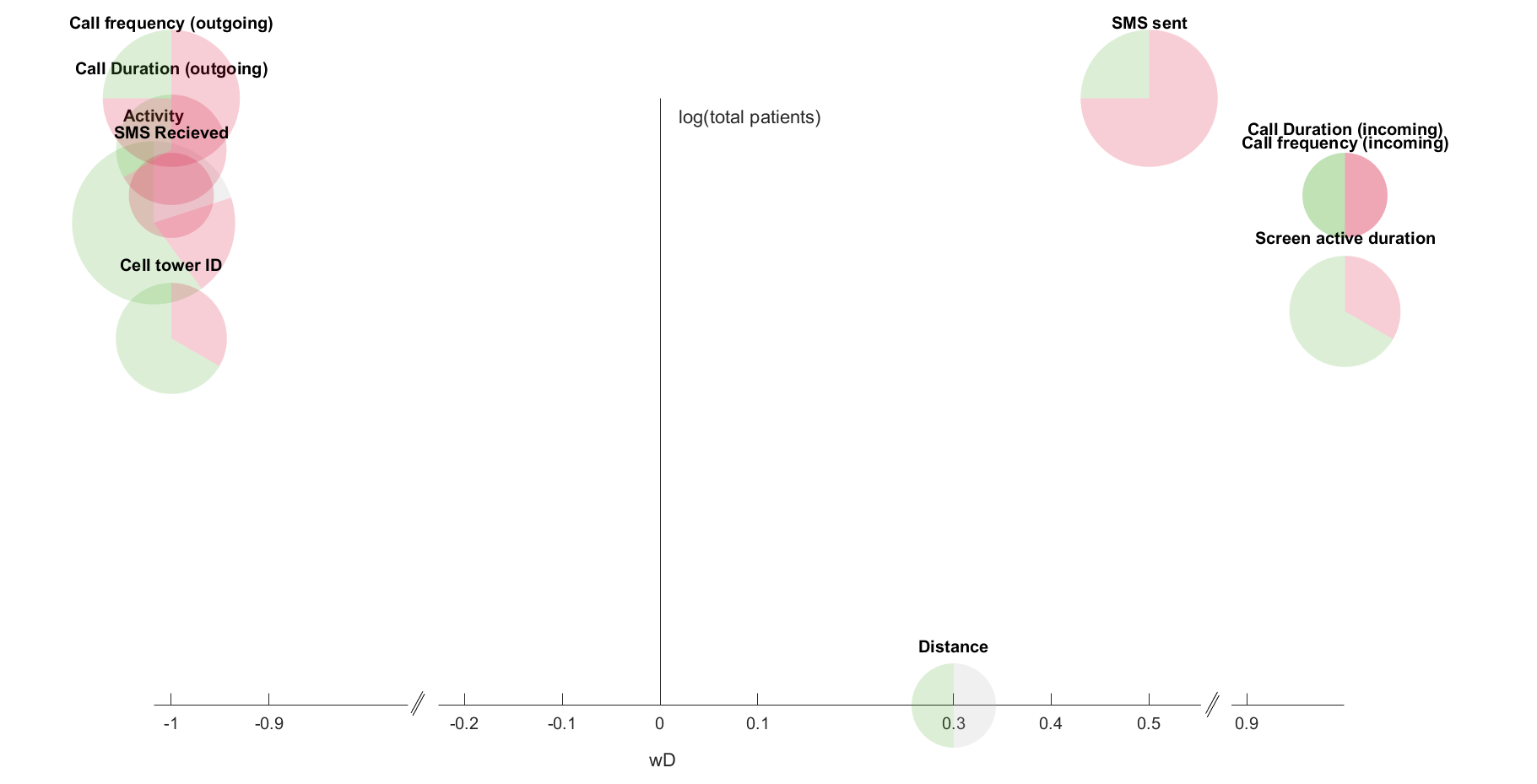

Supplement: Multimedia Appendix 6 [file mhealth_v6i8e165_app6.png]

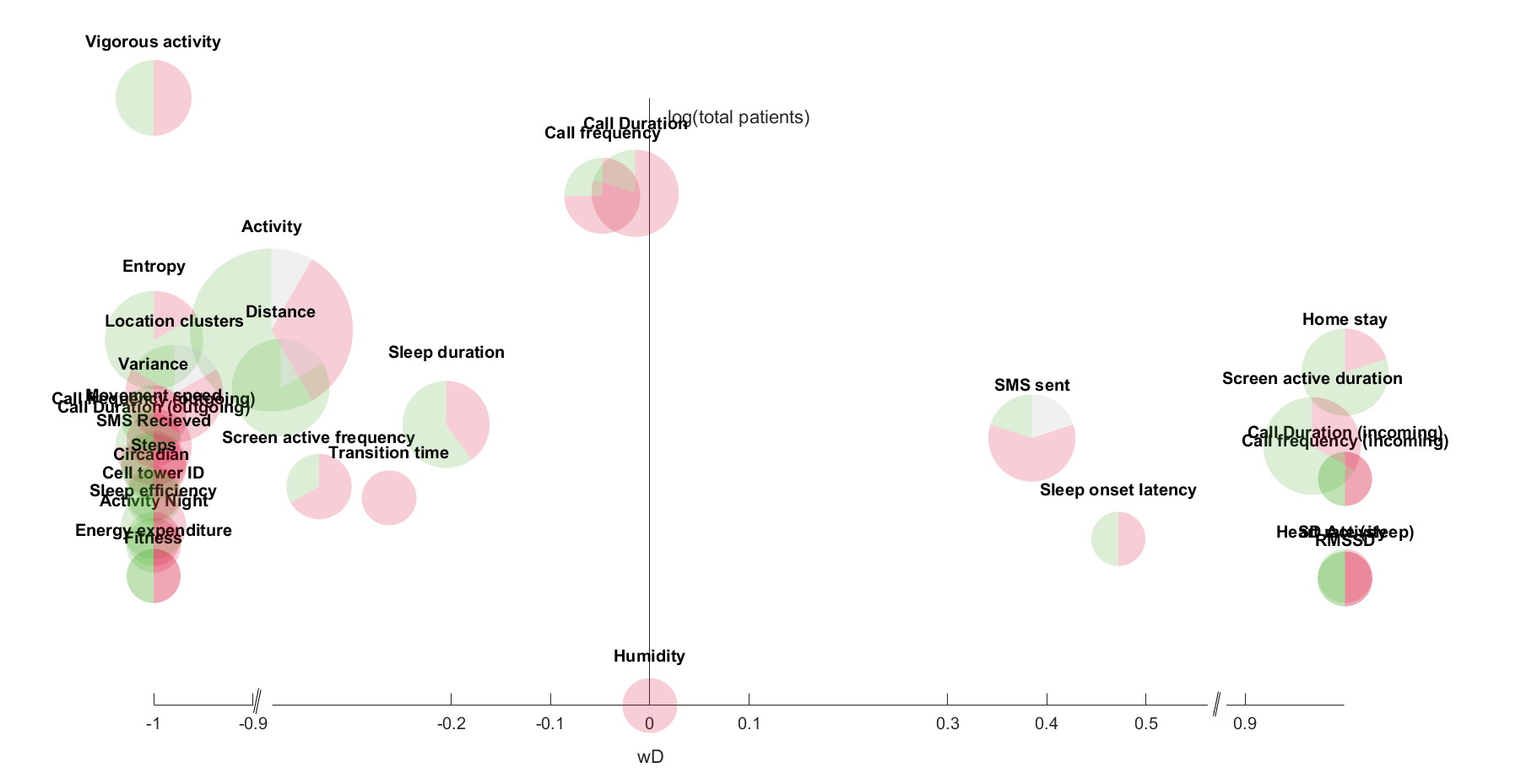

Supplement: Multimedia Appendix 7 [file mhealth_v6i8e165_app7.png]

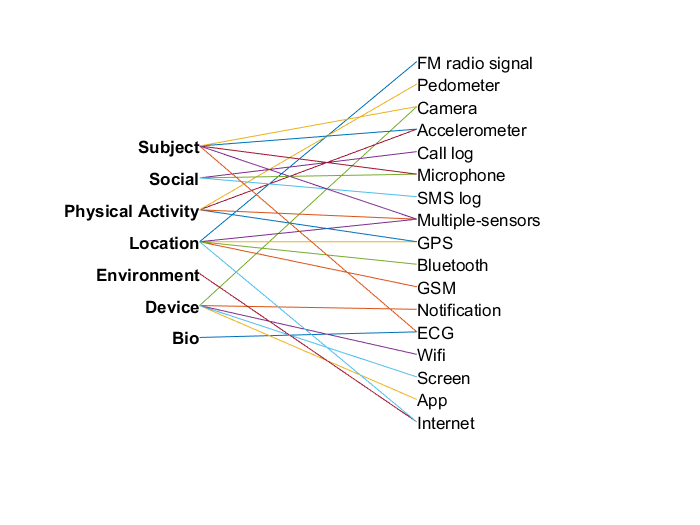

Supplement: Multimedia Appendix 9 [file mhealth_v6i8e165_app9.png]
