# Supplementary material for: Correlations Between Objective Behavioral Features Collected From Mobile and Wearable Devices and Depressive Mood Symptoms in Patients With Affective Disorders: Systematic Review
Source: JMIR Mhealth Uhealth. 2018 Aug 13;6(8):e165. doi: 10.2196/mhealth.9691 (PMC6111148; doi:10.2196/mhealth.9691)
Supplement: Multimedia Appendix 8 [file mhealth_v6i8e165_app8.pdf]

	Physical activity	Social	Location	Subject	Device	Bio	Environment
Abdullah [1]	2	1	1	0	0	0	0
Alvarez-Lozano [2]	0	0	0	0	4	0	0
Asselbergs [3]	1	0	0	0	3	0	0
Baras [4]	0	2	0	0	0	0	0
Becker [5]	1	0	0	0	0	0	0
Beiwinkel [6]	2	3	1	0	0	0	0
Ben-Zeev [7]	1	1	1	1	0	0	0
Berke [8]	0	1	0	0	0	0	0
Berle [9]	2	0	0	0	0	0	0
Canzian [10]	1	0	6	0	0	0	0
Cho [11]	0	4	0	0	0	0	0
Chow [12]	0	0	1	0	0	0	0
DeMasi [13]	1	0	0	1	0	0	0
Dickerson [14]	0	0	0	2	0	0	0
Doryab [15]	0	1	1	0	0	0	2
Edwards [16]	1	0	0	0	0	0	0
Farhan [17]	5	0	5	0	0	0	0
Faurholt-Jepsen [18]	0	2	1	0	1	0	0
Faurholt-Jepsen [19]	0	0	0	1	0	0	0
Faurholt-Jepsen [20]	1	0	0	1	0	1	0
Faurholt-Jepsen [21]	0	10	1	0	2	0	0
Faurholt-Jepsen [22]	2	0	0	1	0	1	0
Faurholt-Jepsen [23]	0	6	0	0	0	0	0
Gershon [24]	1	0	0	0	0	0	0
Gonzalez [25]	2	0	0	0	0	0	0

Grünerbl [26]	1	1	1	1	0	0	0
Guidi [27]	0	0	0	1	0	0	0
Hauge [28]	5	0	0	0	0	0	0
Krane-Gartiser [29]	3	0	0	0	0	0	0
Loprinzi [30]	1	0	0	0	0	0	0
Mark [31]	1	0	0	1	0	0	0
Matic [32]	1	0	1	0	0	0	0
Mehrotra [33]	0	0	0	0	9	0	0
Mestry [34]	0	1	1	0	3	0	0
Miwa [35]	0	0	0	2	0	0	0
Muaremi [36]	0	2	0	3	0	0	0
O'Brien [37]	3	0	0	0	0	0	0
Osmani [38]	3	0	0	0	0	0	0
Palmius [39]	0	0	1	0	0	0	0
Pillai[40]	0	0	0	3	0	0	0
Saeb [41]	1	0	7	0	2	0	0
Saeb [42]	3	0	8	0	0	0	0
St-Amand [43]	1	0	0	4	0	0	0
Todder [44]	2	0	0	0	0	0	0
Wang [45]	0	0	0	1	0	0	0
Wang [46]	0	3	1	1	0	0	0

1. Abdullah S, Matthews M, Frank E, Doherty G, Gay G, Choudhury T. Automatic detection of social rhythms in bipolar disorder. J Am Med Informatics Assoc 2016;23(3):538–543. PMID:26977102
2. Alvarez-Lozano J, Osmani V, Mayora O, Frost M, Bardram J, Faurholt-Jepsen M, Kessing LV. Tell me your apps and I will tell you your mood: correlation of apps usage with bipolar disorder state. Proc 7th Int Conf PErvasive Technol Relat to Assist Environ 2014. p. 19.
3. Asselbergs J, Ruwaard J, Ejdys M, Schrader N, Sijbrandij M, Riper H. Mobile Phone-Based Unobtrusive Ecological Momentary Assessment of Day-to-Day Mood: An Explorative Study. J Med Internet Res [Internet] 2016;18(3):e72. PMID:27025287
4. Baras K, Soares L, Paulo N, Barros R. "Smartphine": Supporting students' well-being according to their calendar and mood. 2016 Int Multidiscip Conf Comput Energy Sci Split 2016 2016; [doi: 10.1109/SpliTech.2016.7555919]
5. Becker D, Bremer V, Funk B, Asselbergs J, Riper H, Ruwaard J. How to Predict Mood? Delving into Features of Smartphone-Based Data. Twenty-second Am Conf Inf Syst 2016;1–10.
6. Beiwinkel T, Kindermann S, Maier A, Kerl C, Moock J, Barbian G, Rössler W. Using Smartphones to Monitor Bipolar Disorder Symptoms: A Pilot Study. JMIR Ment Heal [Internet] 2016;3(1):e2.

PMID:26740354
7. Ben-Zeev D, Scherer EA, Wang R, Xie H, Campbell AT. Next-Generation Psychiatric Assessment: Using Smartphone Sensors to Monitor Behavior and Mental Health HHS Public Access. Psychiatr Rehabil J [Internet] 2015 [cited 2017 Feb 4];38(3):218–226. PMID:25844912
8. Berke EM, Choudhury T, Ali S, Rabbi M. Objective measurement of sociability and activity: Mobile sensing in the community. Ann Fam Med 2011;9(4):344–350. PMID:21747106
9. Berle JO, Hauge ER, Oedegaard KJ, Holsten F, Fasmer OB. Actigraphic registration of motor activity reveals a more structured behavioural pattern in schizophrenia than in major depression. BMC Res Notes 2010;3. PMID:20507606
10. Canzian L, Musolesi M. Trajectories of Depression : Unobtrusive Monitoring of Depressive States by means of Smartphone Mobility Traces Analysis. Proc 2015 ACM Int Jt Conf Pervasive Ubiquitous Comput 2015;1293–1304. [doi: 10.1145/2750858.2805845]
11. Cho YM, Lim HJ, Jang H, Kim K, Choi JW, Shin C, Lee SK, Kwon JH, Kim N. A cross-sectional study of the association between mobile phone use and symptoms of ill health. 2016;1–7.
12. Chow PI, Fua K, Huang Y, Bonelli W, Xiong H, Barnes LE, Teachman BA. Using Mobile Sensing to Test Clinical Models of Depression, Social Anxiety, State Affect, and Social Isolation Among College Students. J Med Internet Res [Internet] 2017;19(3):e62. PMID:28258049
13. Demasi O, Aguilera A, Recht B. Detecting Change in Depressive Symptoms from Daily Wellbeing Questions , Personality , and Activity. 2016;22–29.
14. Dickerson RF, Gorlin EI, Stankovic JA. Empath: a continuous remote emotional health monitoring system for depressive illness. Proc 2nd Conf Wirel Heal - WH '11 [Internet] 2011;Art. 5. [doi: 10.1145/2077546.2077552]
15. Doryab A, Min JK, Wiese J, Zimmerman J, Hong JI. Detection of behavior change in people with depression. AAAI Work Work Twenty-Eighth AAAI Conf Artif Intell 2014;12–16.
16. Edwards MK, Loprinzi PD. Effects of a Sedentary Behavior–Inducing Randomized Controlled Intervention on Depression and Mood Profile in Active Young Adults. Mayo Clin Proc [Internet] 2016 [cited 2017 May 11];91(8):984–998. PMID:27492908
17. Farhan AA, Yue C, Morillo R, Ware S, Lu J, Bi J, Kamath J, Russell A, Bamis A, Wang B. Behavior vs . Introspection : Refining prediction of clinical depression via smartphone sensing data. 2016;30–37.
18. Faurholt-Jepsen M, Frost M, Vinberg M, Christensen EM, Bardram JE, Kessing LV. Smartphone data as objective measures of bipolar disorder symptoms. Psychiatry Res [Internet] 2014 [cited 2017 Feb 5];217(1–2):124–127. PMID:24679993
19. Faurholt-Jepsen M, Busk J, Frost M, Vinberg M, Christensen EM, Winther O, Bardram JE, Kessing L V. Voice analysis as an objective state marker in bipolar disorder. Transl Psychiatry [Internet] 2016 [cited 2017 Feb 5];6(7):e856. PMID:27434490
20. Faurholt-Jepsen M, Brage S, Vinberg M, Jensen HM, Christensen EM, Knorr U, Kessing LV. Electronic monitoring of psychomotor activity as a supplementary objective measure of depression severity. Nord J Psychiatry 2015;69(2):118–125. PMID:25131795
21. Faurholt-Jepsen M, Vinberg M, Frost M, Debel S, Margrethe Christensen E, Bardram JE, Kessing LV. Behavioral activities collected through smartphones and the association with illness activity in bipolar disorder. Int J Methods Psychiatr Res 2016; PMID:21516187
22. Faurholt-jepsen M, Brage S, Vinberg M, Margrethe E, Knorr U, Mørch H, Vedel L. Differences in psychomotor activity in patients suffering from unipolar and bipolar affective disorder in the remitted or mild / moderate depressive state. J Affect Disord [Internet] Elsevier B.V.; 2012;141(2–3):457–463. [doi: 10.1016/j.jad.2012.02.020]
23. Faurholt-Jepsen M, Vinberg M, Frost M, Christensen EM, Bardram JE, Kessing LV. Smartphone data as an electronic biomarker of illness activity in bipolar disorder. Bipolar Disord [Internet] 2015 [cited 2017 Feb 5];17(7):715–728. PMID:26395972
24. Gershon A, Ram N, Johnson SL, Harvey AG, Zeitzer JM. Daily actigraphy profiles distinguish

depressive and interepisode states in bipolar disorder. Clin Psychol Sci 2016;4(4):641–650. PMID:24655651
25. Gonzalez R, Tamminga CA, Tohen M, Suppes T. The relationship between affective state and the rhythmicity of activity in bipolar disorder. J Clin Psychiatry 2014;75(4):1–14. PMID:24500063
26. Grünerbl A, Muaremi A, Osmani V, Bahle G, Öhler S, Tröster G, Mayora O, Haring C, Lukowicz P. Smart-Phone Based Recognition of States and State Changes in Bipolar Disorder Patients. IEEE J Biomed Heal Informatics [Internet] 2015;19(1):140–148. PMID:25073181
27. Guidi A, Vanello N, Bertschy G, Gentili C, Landini L, Scilingo EP. Automatic analysis of speech F0 contour for the characterization of mood changes in bipolar patients. Biomed Signal Process Control [Internet] 2015 [cited 2017 Feb 4];17:29–37. [doi: 10.1016/j.bspc.2014.10.011]
28. Hauge ER, Berle JØ, Oedegaard KJ, Holsten F, Fasmer OB. Nonlinear analysis of motor activity shows differences between schizophrenia and depression: A study using fourier analysis and sample entropy. PLoS One 2011;6(1):1–10. PMID:21297977
29. Krane-Gartiser K, Henriksen TEG, Morken G, Vaaler A, Fasmer OB. Actigraphic assessment of motor activity in acutely admitted inpatients with bipolar disorder. PLoS One 2014;9(2). PMID:24586883
30. Loprinzi PD, Mahoney S. Concurrent occurrence of multiple positive lifestyle behaviors and depression among adults in the United States. J Affect Disord [Internet] Elsevier; 2014;165:126–130. [doi: 10.1016/j.jad.2014.04.073]
31. Mark G, Czerwinski M, Iqbal S, Johns P. Workplace Indicators of Mood: Behavioral and Cognitive Correlates of Mood Among Information Workers. Proc 6th Int Conf Digit Heal Conf [Internet] 2016;29–36. [doi: 10.1145/2896338.2896360]
32. Matic A, Osmani V, Popleteev A, Mayora-Ibarra O. Smart phone sensing to examine effects of social interactions and non-sedentary work time on mood changes. Lect Notes Comput Sci (including Subser Lect Notes Artif Intell Lect Notes Bioinformatics) 2011;6967 LNAI:200–213. [doi: 10.1007/978-3-642-24279-3_21]
33. Mehrotra A, Hendley R, Musolesi M. Towards multi-modal anticipatory monitoring of depressive states through the analysis of human-smartphone interaction. Proc 2016 ACM Int Jt Conf Pervasive Ubiquitous Comput Adjun - UbiComp '16 [Internet] 2016 [cited 2017 Feb 5]. p. 1132–1138. [doi: 10.1145/2968219.2968299]
34. Mestry M, Mehta J, Mishra A, Gawande K. Identifying associations between smartphone usage and mental health during depression, anxiety and stress. Proc - 2015 Int Conf Commun Inf Comput Technol ICCICT 2015 2015; [doi: 10.1109/ICCICT.2015.7045656]
35. Miwa H, Sasahara S, Matsui T. Roll-over detection and sleep quality measurement using a wearable sensor. Conf Proc IEEE Eng Med Biol Soc [Internet] IEEE; 2007 Aug [cited 2017 Feb 4];2007:1507–1510. PMID:18002253
36. Muaremi A, Gravenhorst F, Grünerbl A, Arnrich B, Tröster G. Assessing bipolar episodes using speech cues derived from phone calls. Lect Notes Inst Comput Sci Soc Telecommun Eng LNICST 2014. p. 103–114. [doi: 10.1007/978-3-319-11564-1_11]
37. O'Brien JT, Gallagher P, Stow D, Hammerla N, Ploetz T, Firbank M, Ladha C, Ladha K, Jackson D, McNaney R, Ferrier IN, Olivier P. A study of wrist-worn activity measurement as a potential real-world biomarker for late-life depression. Psychol Med [Internet] 2016;No-Specified. PMID:27667663
38. Osmani V, Maxhuni A, Grünerbl A, Lukowicz P, Haring C, Mayora O. Monitoring Activity of Patients with Bipolar Disorder Using Smart Phones. Proc Int Conf Adv Mob Comput & Multimed [Internet] 2013. p. 85:85--85:92. [doi: 10.1145/2536853.2536882]
39. Palmius N, Tsanas A, Saunders KEA, Bilderbeck AC, Geddes JR, Goodwin GM, De Vos M. Detecting Bipolar Depression from Geographic Location Data. IEEE Trans Biomed Eng [Internet] 2016 [cited 2017 Feb 6];1–1. [doi: 10.1109/TBME.2016.2611862]
40. Pillai V, Steenburg LA, Ciesla JA, Roth T, Drake CL. A seven day actigraphy-based study of rumination and sleep disturbance among young adults with depressive symptoms. J Psychosom Res [Internet]

Elsevier Inc.; 2014;77(1):70–75. PMID:24913345
41. Saeb S, Zhang M, Karr CJ, Schueller SM, Corden ME, Kording KP, Mohr DC. Mobile phone sensor correlates of depressive symptom severity in daily-life behavior: An exploratory study. J Med Internet Res 2015;17(7):1–11. PMID:26180009
42. Saeb S, Lattie EG, Schueller SM, Kording KP, Mohr DC. The relationship between mobile phone location sensor data and depressive symptom severity. PeerJ [Internet] 2016;4:e2537. PMID:28344895
43. St-Amand J, Provencher MD, Bélanger L, Morin CM. Sleep disturbances in bipolar disorder during remission. J Affect Disord 2013;146(1):112–119. PMID:22884237
44. Todder D, Caliskan S, Baune BT. Longitudinal changes of day-time and night-time gross motor activity in clinical responders and non-responders of major depression. World J Biol Psychiatry 2009;10(4):276–284. PMID:19921969
45. Wang R, Campbell AT, Zhou X. Using opportunistic face logging from smartphone to infer mental health. Proc 2015 ACM Int Jt Conf Pervasive Ubiquitous Comput Proc 2015 ACM Int Symp Wearable Comput - UbiComp '15 [Internet] 2015;683–692. [doi: 10.1145/2800835.2804391]
46. Wang R, Chen F, Chen Z, Li T, Harari G, Tignor S, Zhou X, Ben-Zeev D, Campbell AT. StudentLife: assessing mental health, academic performance and behavioral trends of college students using smartphones. Proc 2014 ACM Int Jt Conf Pervasive Ubiquitous Comput 2014;3–14. [doi: 10.1145/2632048.2632054]
